# Using Machine Learning for the Discovery and Development of Multitarget Flavonoid-Based Functional Products in MASLD

**DOI:** 10.3390/molecules30214159

**Published:** 2025-10-22

**Authors:** Maksim Kuznetsov, Evgeniya Klein, Daria Velina, Sherzodkhon Mutallibzoda, Olga Orlovtseva, Svetlana Tefikova, Dina Klyuchnikova, Igor Nikitin

**Affiliations:** 1 Department of Food Technology and Bioengineering, Plekhanov Russian University of Economics, 36 Stremyanny per., 115054 Moscow, Russia; mutallibzoda@bk.ru (S.M.); orlovtseva.oa@rea.ru (O.O.); tefikova.sn@rea.ru (S.T.); klyuchnikova.dv@rea.ru (D.K.); 2 Research Laboratory “Biotechnology of Food Systems”, Plekhanov Russian University of Economics, 36 Stremyanny per., 115054 Moscow, Russia; kattim67@gmail.com

**Keywords:** MASLD, nutraceutical design, in silico screening, machine learning, flavonoids, PBPK modeling, multi-target strategy, chemical space clustering

## Abstract

Metabolic dysfunction-associated steatotic liver disease (MASLD) represents a multifactorial condition requiring multi-target therapeutic strategies beyond traditional single-marker approaches. In this work, we present a fully in silico nutraceutical screening pipeline that integrates molecular prediction, systemic aggregation, and technological design. A curated panel of ten MASLD-relevant targets, spanning nuclear receptors (FXR, PPAR-α/γ, THR-β), lipogenic and cholesterogenic enzymes (ACC1, FASN, DGAT2, HMGCR), and transport/regulatory proteins (LIPG, FABP4), was assembled from proteomic evidence. Bioactivity records were extracted from ChEMBL, structurally standardized, and converted into RDKit descriptors. Predictive modeling employed a stacked ensemble of Random Forest, XGBoost, and CatBoost with isotonic calibration, yielding robust performance (mean cross-validated ROC-AUC 0.834; independent test ROC-AUC 0.840). Calibrated probabilities were aggregated into total activity (TA) and weighted TA metrics, combined with structural clustering (six structural clusters, twelve MOA clusters) to ensure chemical diversity. We used physiologically based pharmacokinetic (PBPK) modeling to translate probabilistic profiles into minimum simulated doses (MSDs) and chrono-specific exposure (%T>IC50) for three prototype concepts: HepatoBlend (morning powder), LiverGuard Tea (evening aqueous form), and HDL-Chews (postprandial chew). Integration of physicochemical descriptors (MW, logP, TPSA) guided carrier and encapsulation choices, addressing stability and sensory constraints. The results demonstrate that a computationally integrated pipeline can rationally generate multi-target nutraceutical formulations, linking molecular predictions with systemic coverage and practical formulation specifications, and thus provides a transferable framework for MASLD and related metabolic conditions.

## 1. Introduction

The term metabolic dysfunction-associated steatotic liver disease (MASLD) has replaced the designation “non-alcoholic fatty liver disease” (NAFLD), in use since 1980, which was deemed inadequate due to its pejorative tone and reliance on exclusionary diagnostic criteria (absence of excessive alcohol consumption) rather than the metabolically meaningful factors underlying hepatic steatosis [1]. This revision of terminology and nosology has materially improved clinical practice in diagnosing steatotic liver disease by helping overcome instrumental and biochemical limitations such as the lack of specific early-stage symptoms, the high cost of effective imaging modalities (e.g., MRI), and the invasiveness and risk of liver biopsy—the conventional procedure for identifying NAFLD. The new diagnostic criteria emphasize blood-based biomarkers of steatosis and shift the focus away from “absence of alcohol” toward the active detection of metabolic derangements, including overweight, obesity, type 2 diabetes (T2D), hypertension, insulin resistance, hypercholesterolemia, hyperglycemia, dyslipidemia, and elevated hs-CRP [2].

An important positive consequence of the transition from NAFLD to MASLD has been the consolidation of an interdisciplinary approach to diagnosis and therapy: involvement of cardiologists, endocrinologists, and dietitians has broadened coverage to more patients with steatosis, including lean individuals [3]. Notably, the overwhelming majority of patients previously diagnosed with NAFLD meet current MASLD criteria (94.5–98%), whereas a comparatively smaller proportion of MASLD patients meet NAFLD criteria (84.1%) [3,4]. This both affirms the relevance of the clinical evidence accumulated over the past four decades under the updated nomenclature and supports the utility of the new definitions for improving risk management related to liver health.

Despite advances in diagnostic strategies, the global burden of hepatic steatosis remains substantial and demands continued development of new preventive and therapeutic avenues. Epidemiological data indicate a marked increase in MASLD prevalence: while in 1991 the condition affected 22% of the adult population worldwide, during 2019–2024, global adult prevalence was estimated to be at 37–38% [2,5], reaching 47.2% in some regions (e.g., South Korea) [3]. Individuals with obesity are particularly vulnerable, with MASLD prevalence up to 75.3% [6], primarily reflecting disturbances in lipid metabolism and hepatic triglyceride accumulation.

In current understanding, MASLD is not an isolated liver disorder; rather, it denotes impairment of hepatic function against a backdrop of systemic metabolic dysfunction. Its pathogenesis reflects a tightly interwoven constellation of factors that interact and evolve as the disease progresses. Hyperinsulinemia and adipose tissue insulin resistance stimulate lipolysis and augment hepatic gluconeogenesis and lipogenesis, overloading hepatocytes with free fatty acids (FFAs) [7]. Likewise, a sustained caloric surplus from dietary fats and carbohydrates (≥10% above energy expenditure) increases FFA influx to the liver [6]. Lipotoxic FFA excess triggers endoplasmic reticulum stress and mitochondrial dysfunction, accompanied by reactive oxygen species (ROS) accumulation and oxidative injury to cellular structures [8]. These processes activate Kupffer cells (resident hepatic macrophages) and recruit monocytes, with involvement of T-lymphocytes and neutrophils that amplify inflammation and tissue damage. Under chronic inflammation, hepatic stellate cells are activated, leading to progressive fibrosis that can culminate in cirrhosis and hepatocellular carcinoma [9]. Dysregulated bile acid homeostasis and gut microbiota imbalance also contribute materially by increasing intestinal permeability and inducing pro-inflammatory mediators [7]. Consequently, effective MASLD therapy must address multiple targets simultaneously—attenuating insulin resistance, oxidative stress, mitochondrial dysfunction, and dysbiosis in parallel.

Contemporary omics studies underscore that MASLD signatures arise from coordinated shifts in the transcriptome and proteome, encompassing receptor-level sensors (e.g., FXR), regulators of fatty acid catabolism (PPAR-α), and enzymes of de novo lipogenesis (ACC1/FASN/DGAT2) [10,11,12,13]. The functional significance of these targets is briefly outlined below.

FXR (Farnesoid X receptor), a member of the nuclear-receptor superfamily, plays a central role in fatty acid and glucose metabolism and in the biosynthesis, utilization, and transport of triglycerides; FXR activation is therefore widely leveraged to reduce hepatocellular burden in MASLD therapy [14].

PPAR-α (peroxisome proliferator-activated receptor alpha), also a nuclear receptor, is a key transcriptional regulator of lipid metabolism. It governs the expression of numerous genes involved in fatty acid uptake and mitochondrial β-oxidation, gluconeogenesis, triglyceride handling, and inflammatory signaling [13,15]. Reduced hepatic PPAR-α expression has been observed in MASLD patients [15], underscoring its therapeutic relevance while not limiting the scope of medical intervention.

De novo lipogenesis denotes the synthesis of fatty acids from carbohydrates, predominantly glucose. The resulting fatty acids may be esterified into triglycerides and accumulate in the liver; in MASLD, this pathway contributes up to 26% of hepatocellular triglycerides [16]. Key enzymes include ACC1 (acetyl-CoA carboxylase 1), which converts acetyl-CoA to malonyl-CoA, the substrate for fatty acid synthesis; FASN (fatty acid synthase), which produces long-chain fatty acids; and DGAT2 (diacylglycerol O-acyltransferase 2), which catalyzes triglyceride formation from diacylglycerols and fatty acids. Inhibitors of these enzymes are promising MASLD therapeutics and have been shown in vivo and in vitro to reduce hepatic fat and triglyceride content and exert antifibrotic effects [17,18].

Additional proteins whose activity has been implicated in MASLD and related metabolic disturbances merit consideration as therapeutic targets. These include HMGCR (3-hydroxy-3-methylglutaryl-CoA reductase), a key enzyme in cholesterol biosynthesis and a potential mediator at the lipid–carbohydrate interface [19]; FABP4 (fatty acid-binding protein 4), whose increased expression has been observed in hepatocytes in both alcoholic and non-alcoholic steatohepatitis [20]; and LIPG (endothelial lipase), involved in lipoprotein hydrolysis and a translational candidate in obesity prevention [21].

As inhibitors or activators of these targets, naturally derived bioactives—especially flavonoids—are increasingly employed. Multiple studies have shown that flavonoids upregulate PPAR-α expression, bolster endogenous antioxidant defenses, inhibit the NF-κB inflammatory pathway and pro-inflammatory enzymes, modulate the gut microbiota, and regulate the expression of genes involved in de novo lipogenesis and fatty acid oxidation [22,23,24].

Over the past decade, machine learning has progressed from classical QSAR to deep models that can discover novel chemotypes and prioritize bioactives at scale. For example, a deep neural network trained on structure–activity data prospectively identified halicin and other antibiotics, illustrating the ability of ML to generalize beyond known scaffolds. Structure-aware graph neural networks now set strong baselines for drug–target affinity prediction (e.g., GraphDTA and related GNN variants), improving upon fixed fingerprints by learning molecular graphs and protein representations [25]. In parallel, a growing body of work integrates ML with PBPK to predict ADME/PK parameters and human exposure, reducing experimental burden while keeping physiological interpretability; recent reviews and studies outline practical ML → PBPK workflows and demonstrate accurate PK parameter prediction [26]. For natural products, AI increasingly supports dereplication, target prediction, and multi-property screening in complex mixtures, accelerating discovery from botanical sources [27]. Within this context, our calibrated ensemble models, chemical-space mapping, and screening-level PBPK provide a transparent, reproducible bridge from multitarget likelihoods to exposure-linked formulation hypotheses, aligned with current best practices.

Here, we present a comprehensive in silico approach to multitarget screening of flavonoid-based nutraceutical candidates for MASLD therapy, integrating systems biology data, chemical information, and modern machine learning methods. The pipeline encompasses assembly of a panel of key biomolecular targets; standardized extraction and curation of compound bioactivity data; generation of chemical descriptors; construction and calibration of ensemble predictive models; multitarget candidate ranking with pathway-aware weighting; and evaluation of hepatic behavior using PBPK modeling. This approach systematically links molecular properties to physiological context and to the technological aspects of nutraceutical design.

## 2. Results

### 2.1. Model Performance

The constructed ensemble—comprising Random Forest, XGBoost, and CatBoost as base learners, with second-level logistic meta-regression and isotonic calibration—achieved robust class separation across all ten MASLD targets in a fully in silico setting. Under Murcko- and time-split cross-validation, the mean ROC-AUC was 0.834 (median = 0.840; range = 0.750–0.916). On independent test sets, the mean test ROC-AUC was 0.840 (median = 0.832; range = 0.758–0.956). The strongest performance was observed for CHEMBL2083 (FABP4, 0.956), CHEMBL1947 (0.930), and CHEMBL3247 (HMGCR, 0.882), while the weakest results were obtained for DGAT2 (0.758), FXR (0.765), and PPAR-γ (0.774).

Panel-averaged metrics further supported ensemble adequacy under class imbalance: F1 = 0.719 (median = 0.732) and MCC = 0.507 (median = 0.499).

Concordance among the base models in ranking the prioritized candidate pool was consistently high. The global Kendall’s W coefficient reached 0.829, with per-target values ranging from 0.558 to 0.924, reflecting a mixture of “easier” and more variable tasks. The mean pairwise Jaccard index across top-N lists was 0.716, capturing a balance between overlapping leads and model complementarity.

Isotonic calibration ensured reliable probabilistic interpretation: the overall Brier score averaged 0.094 (median = 0.088; range = 0.018–0.150), with the lowest calibration error observed for CHEMBL4158 and the highest for CHEMBL235. The Hosmer–Lemeshow test indicated no evidence of miscalibration (HL-p spanning 0.995–1.000; mean = 0.999), further confirming robustness of probability estimates.

Unless otherwise specified in subsequent sections, the binary classification threshold is fixed at *p* ≥ 0.50, as defined in the pipeline configuration. All conclusions are derived from internal splits and time-resampling schemes without laboratory validation and thus remain strictly within the in silico framework.

### 2.2. Chemical Space Mapping and Probabilistic Heat Map

Structural stratification of the prioritized pool was performed using Morgan fingerprints (ECFP4, radius = 2; 2048 bits) with the Tanimoto similarity metric. The clustering cut-off was determined in auto_cut mode across a candidate grid of 0.10–0.40, selecting the threshold that maximized the silhouette score; in the present run, thr = 0.20 was chosen. The resulting topology at the structural level comprised six clusters with size distribution [32, 1, 1, 1, 1, 1], i.e., one dominant “core” and five singletons. This partitioning reflects that, under a relatively permissive threshold (0.20), chemically diverse polyphenolic and terpenoid motifs were drawn into the central core, while rare scaffolds remained as isolated nodes.

In parallel, mechanism-of-action (MOA) clusters were generated from probability vectors *p*(target), using cosine similarity with auto-thresholding and kNN fallback. This produced 12 clusters with sizes [9, 6, 5, 5, 3, 2, 2, 1 × 5]; medoids from these clusters were subsequently used in portfolio selection.

**Figure 1:**
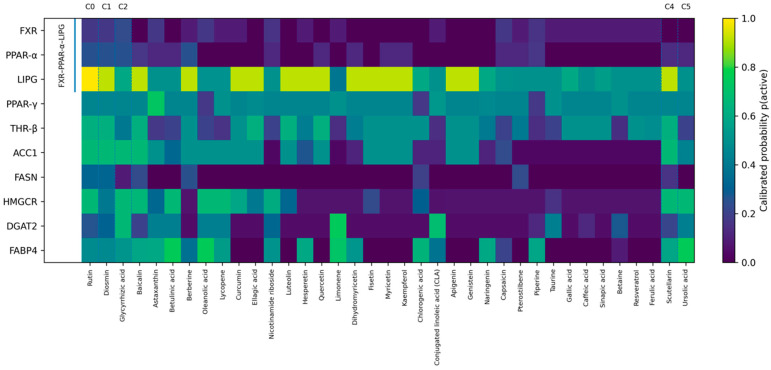
Target × compound probabilistic heat-map.

Figure 1 was constructed from all 37 candidates. Columns were ordered exactly as in the postprocessing script: first by cluster_id (ascending), then by TA_weighted (descending), and finally by main_score (descending). For each target, calibrated probabilities *p*(active) are displayed. Selectivity markers (criterion: max *p* ≥ 0.80 and second max *p* ≤ 0.40) were not assigned in this run, as no compound met these thresholds.

To diagnose local SAR discontinuities, break-gap scanning was applied: the base mask (Tanimoto ≥ 0.80 and Δ*p* ≥ 0.40) revealed a single pair with a sharp probability drop (for target LIPG). Extended scanning across the grid (tan_thr ∈ {0.75, 0.80, 0.85}; gap_thr ∈ {0.30, 0.40, 0.50}) returned additional candidates for manual review, consistent with the “patchy” probability patterns observed within the large structural core.

Consistent co-activation patterns were most evident among target pairs with high rank correlation of probabilities across the 37 compounds: LIPG–THR-β (ρ ≈ 0.77) and LIPG–ACC1 (ρ ≈ 0.73) formed pronounced “combs” of joint elevation; PPAR-α–FASN (ρ ≈ 0.62) and DGAT2–FABP4 (ρ ≈ 0.57) also showed positive associations. At the same time, negative associations (LIPG ↔ DGAT2, THR-β ↔ DGAT2) highlighted the coexistence of multi-target and narrower profiles within the same structural core. Collectively, this landscape provides a foundation for designing formulations with controlled overlap of targets across different chrono-windows.

### 2.3. Formulation Selection and Rationale

Formulation design was conducted entirely in silico, integrating the results of composite ranking (TA/TA_weighted), EFSA/FDA regulatory constraints, and PBPK modeling of hepatic exposure (one-compartment Bateman model with first-order absorption and elimination, parameterized according to the physicochemical profiles of flavonoids) [28,29,30]. For each chrono-window (“morning”, “evening”, “postprandial”), the engineering criterion was target coverage defined as %T>IC50 at IC_50_ = 1 μM for the window’s priority targets.

The PBPK component was implemented as a screening-level one-compartment Bateman-type model with first-order absorption and elimination kinetics. This simplified one-compartment Bateman-type model served as a screening-level tool to estimate exposure (%T>IC50, C_max_) for formulation ranking and should not be interpreted as a full physiologically based PBPK model, which normally includes multiple compartments and organ-specific kinetics. Parameterization of absorption rate constants (Ka) and clearance (Cl) was guided by the compounds’ physicochemical descriptors (molecular weight, logP, and TPSA), following empirical scaling relationships reported in the literature [28,29,30]. This approach provides a reproducible engineering approximation that links predicted probabilities of target activity to feasible dose–exposure ranges. It does not represent a full physiologically based PBPK system, and its outputs serve as indicative estimates for formulation design. The key assumptions and limitations are summarized in Table S1 (“Planned Validation Roadmap”).

The same one-compartment Bateman-type model was consistently applied across all compounds to maintain reproducibility and comparability of exposure profiles. The absorption (Ka) and clearance (Cl) parameters were estimated from empirical in silico relationships linking molecular weight, logP, and TPSA to kinetic behavior, following published pharmacokinetic scaling studies [28,29,30]. This approach provides uniform assumptions across the compound set and will be refined in future in vitro and ex vivo stages (Table S1).

Each chrono-window was simulated independently under static parameterization of absorption and clearance, without intra-day variation in Ka or Cl. This screening-level design isolates formulation timing effects conceptually; future dynamic PBPK versions will incorporate circadian modulation of pharmacokinetic parameters.

The exposure metrics (%T>IC50, C_max_) were derived from screening-level PBPK simulations and are intended solely as engineering estimates linking predicted activity probabilities with feasible dose ranges. These values are not experimentally verified pharmacokinetic measurements and should be interpreted as indicative for formulation design rather than as biological confirmation.

**Figure 2:**
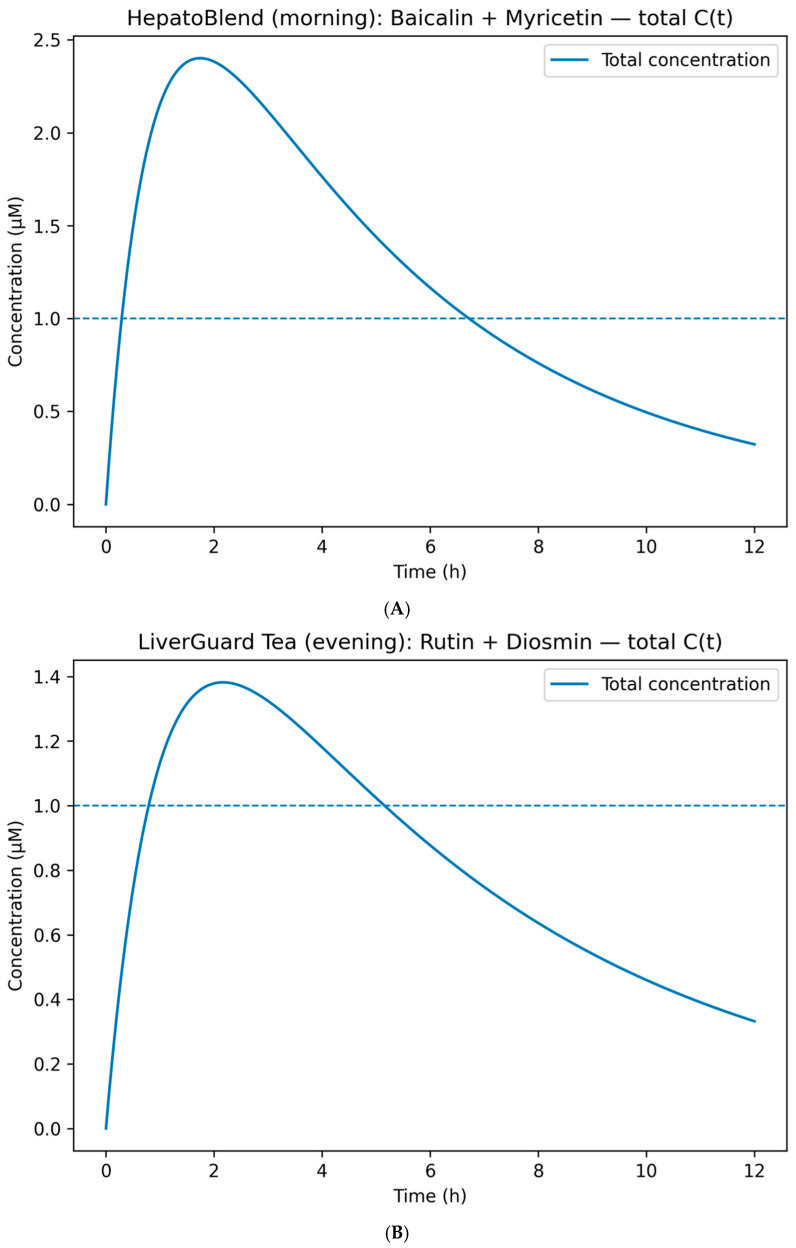

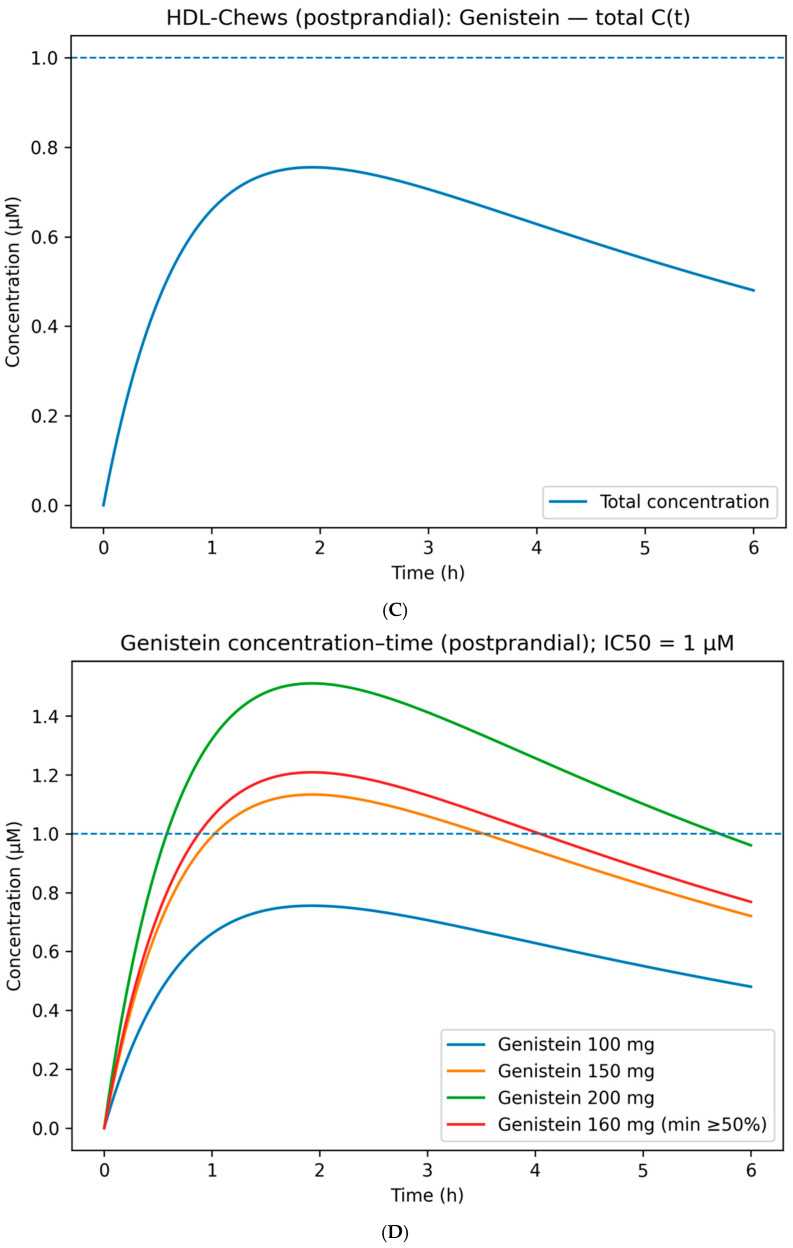

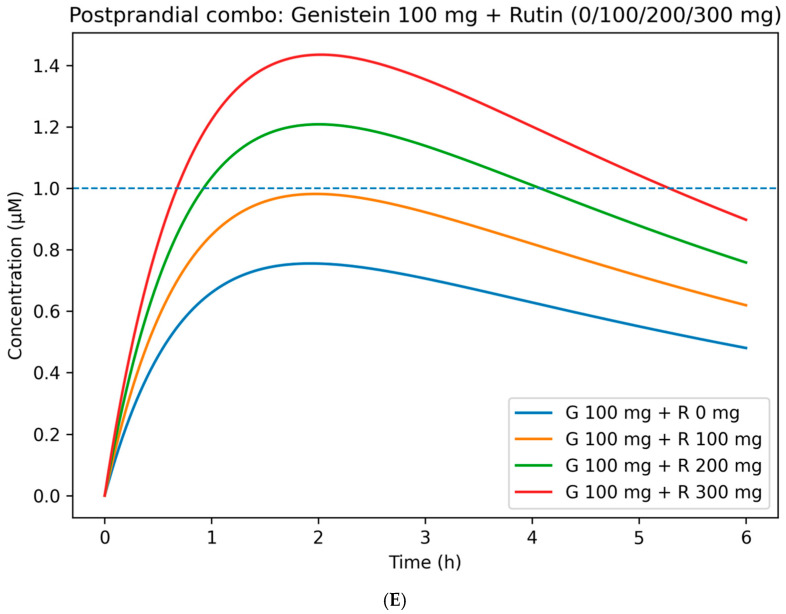
(**A**). HepatoBlend (morning): Baicalin + Myricetin—total C_hep(t). One-compartment Bateman model; the combined C_hep(t) profile of the pair is shown for clarity. The %T>IC50 calculation was performed per target effect (Hill n = 1) and aggregated. C_max ≈ 2.4 μM, %T>IC50(1 μM) ≈ 6–7 h. (**B**). LiverGuard Tea (evening): Rutin + Diosmin—total C_hep(t). Smoothed evening exposure profile; C_max ≈ 1.35–1.40 μM, with concentrations maintained ≳1 μM дo ~6 h. Coverage of the “evening” window targets confirmed by per-target aggregation. (**C**). HDL-Chews (postprandial): Genistein—final dose. Final curve for ≈160 mg; dashed line indicates IC_50_ = 1 μM. Target %T>IC50 ≥ 50% in the 0–6 h interval for LIPG/FABP4. (**D**). Genistein dose finding (100/150/160/200 mg). Increase in C_max (~0.75 → ~1.15/1.20 → ~1.50 μM) and corresponding expansion of %T>IC50. The minimally sufficient dose is ≈160 mg. (**E**). Postprandial combo: Genistein 100 mg + Rutin (0/100/200/300 mg). Systemic effect calculated per target and aggregated (HSA/Bliss models). At 200–300 mg rutin, systemic coverage comparable to the monotherapy regimen of 160–200 mg genistein is achieved, without increasing the genistein dose.

In the plots shown in Figure 2, the dashed line indicates the 1 μM threshold; solid curves represent the simulated total Chep(t) for the selected combination (sum of hepatic concentrations). However, %T>IC50 was not calculated on total concentrations directly but through per-target effect aggregation: for each target *k* and component *i*, the effect was computed as

$$E_{i,k}(t)=\frac{C_{i}(t)}{{IC}_{50,k}+C_{i}(t)\;}$$

(Hill n = 1). These were aggregated across components (HSA/Bliss independence assumption) and then averaged across targets within the chrono-window using window-specific weights. This approach prevented spurious “threshold crossings” that could arise from simple arithmetic addition of different compounds’ concentrations.

All compositions presented below were curated from the auto-generated portfolio: selection was restricted to one member per structural and MOA cluster, ensured coverage of window-specific targets, and incorporated considerations of technological feasibility of the dosage matrix.

HepatoBlend (morning; Baicalin + Myricetin). The morning formulation targets FXR/PPAR-α cascades during the phase of heightened β-oxidation [2]. The cumulative Chep(t) reaches C_max_ ≈ 2.4 μM at t_max_ ~2 h and remains above 1 μM for ~6–7 h (Figure 2A). Per-target aggregation confirms adequate %T>IC50 without dose escalation. A validated alternative, Baicalin + Luteolin, produces a comparable profile and may be substituted when sensory or matrix constraints apply.

LiverGuard Tea (evening; Rutin + Diosmin). The evening formulation provides a smoother profile focused on PPAR-γ and enzymatic recovery pathways [31]. The combined Chep(t) curve shows C_max_ ≈ 1.35–1.40 μM at t_max_ ~2 h, with concentrations maintained ≳1 μM up to ~6 h (Figure 2B). Owing to the slower absorption of glycosides, this profile is compatible with an aqueous (infusion) matrix, and %T>IC50 across evening-window targets is achieved at moderate peak levels.

HDL-Chews (postprandial; Genistein). For the postprandial window, dosing was minimized while maintaining %T>IC50 ≥ 50% over the 0–6 h interval [28]. For the postprandial window, dosing was minimized while maintaining %T>IC50 ≥ 50% over the 0–6 h interval [28]. The baseline 100 mg simulation served as a reference for optimization; corresponding values are described in the PBPK modeling section of Materials and Methods and explicitly labeled as “Baseline (100 mg)” vs. “Optimized regimen” to avoid ambiguity. Dose–response curves (Figure 2D) show that 100 mg is insufficient (C_max_ ~0.75 μM), whereas 150–200 mg achieves concentrations ≥1 μM. The minimally sufficient dose is ≈160 mg (C_max_ ~1.2 μM). The final monotherapy profile is shown in Figure 2C, with %T>IC50 calculated by per-target effect aggregation, primarily addressing LIPG/FABP4 during the postprandial period.

Exposure-enhanced variant (postprandial combination). To reduce the genistein dose, combinations of Genistein 100 mg + Rutin 100/200/300 mg were simulated (Figure 2E). Rutin does not contribute concentration “additively” for the same target but increases systemic probability through complementary and partially overlapping profiles (aggregated using HSA/Bliss independence). At 200–300 mg rutin, systemic coverage comparable to the 160–200 mg genistein monotherapy is achieved, but with a reduced genistein dose—technologically advantageous for chewable formulations (rapid release ≤ 5 min; reduced bitterness and portion mass). Where necessary, absorption can be enhanced via phytosomal complexes (for quercetin, relative bioavailability increases up to ~5.2× have been reported) without altering the underlying coverage logic [32].

Taken together, the calculated profiles demonstrate that: (i) HepatoBlend generates a morning plateau of aglycone exposure with coverage of FXR/PPAR-α; (ii) LiverGuard Tea achieves evening coverage with moderate peaks oriented toward PPAR-γ-related effects; (iii) HDL-Chews meet the postprandial coverage target either as genistein monotherapy (≈160–200 mg) or in combination with rutin at lower genistein doses. All conclusions are in silico and serve as engineering specifications for subsequent experimental validation.

To facilitate interpretation, the comparison between pre-optimization (“Baseline 100 mg”) and optimized (“Final regimen”) PBPK scenarios for each chrono-window is described in the PBPK modeling section of Materials and Methods.

### 2.4. Stability and Sensory Assessment

**Figure 3:**
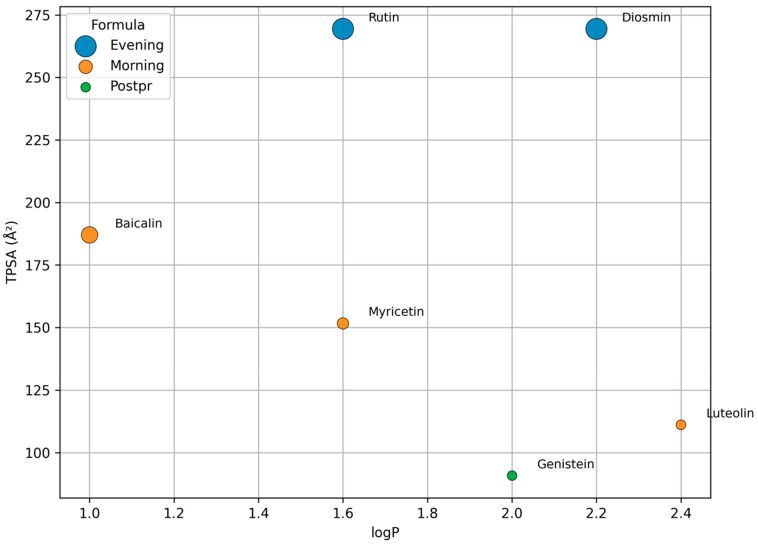
Physicochemical diversity of key actives. Scatter plot of logP vs. TPSA (point size ∝ MW) for Baicalin, Myricetin, Luteolin (Morning), Rutin, Diosmin (Evening), and Genistein (Postprandial). Points are colored by target formulation; lead compounds are annotated.

The in silico-designed formulations, assembled from lead compounds identified through multi-target ranking, were shown to achieve the target pharmacokinetic metric of %T>IC50 while also meeting technological stability and sensory acceptability requirements, provided that carriers and processing regimes were rationally selected. At the predictive stage, calibrated probabilities *p*(active) across the ten MASLD targets were aggregated into composite TA/TA_weighted indices; subsequent filtering rules (TA → main score → TA_weighted → structural diversification) prioritized candidates for the three chrono-windows (“morning”, “evening”, “postprandial”), with results recorded in ranked_hits.csv and portfolio.csv. For technological interpretation, each compound was mapped to key physicochemical descriptors—molecular weight (MW), lipophilicity (logP), and topological polar surface area (TPSA)—which served as a bridge between the probabilistic “target × compound” profiles, hepatic exposure, and engineering decisions regarding carriers, processing/packaging modes, and taste masking. All visualizations in the current report were generated using a unified fallback descriptor set to ensure reproducibility across Figure 3 and Table 1: Baicalin (MW 446.36; logP 1.0; TPSA 187.0), Myricetin (MW 318.24; logP 1.6; TPSA 151.6), Luteolin (MW 286.24; logP 2.4; TPSA 111.1), Rutin (MW 610.52; logP 1.6; TPSA 269.4), Diosmin (MW 608.54; logP 2.2; TPSA 269.4), and Genistein (MW 270.24; logP 2.0; TPSA 90.9). Figure 3 presents a logP–TPSA scatter plot (point size proportional to MW) for these “anchor” actives of the morning, evening, and postprandial formulations, directly linking physicochemical placement with carrier requirements and taste attributes. Table 1 is programmatically synchronized with the same descriptor set, consolidating stability risks, recommended carriers/matrices, and predicted sensory profiles for each compound.

In the morning HepatoBlend (powdered drink) format, the anchor flavones/flavonols—Baicalin, Myricetin, and Luteolin—occupy an intermediate logP–TPSA zone where oxidative and photochemical degradation risks and solubility limitations are simultaneously relevant. For Baicalin, pH-dependent glycosidic hydrolysis, photodegradation, and phenolic oxidation are key risks; mitigation involves glassy microcapsules (maltodextrin/arabic gum), light-impermeable packaging, and maintenance of a mildly acidic microenvironment [33,34]. Myricetin is dominated by poor solubility and a propensity for oxidation/darkening in aqueous systems, justifying solid lipid nanoparticles, self-emulsifying/liposomal systems, or polysaccharide coatings [35,36,37]. Luteolin exhibits poor solubility, photolability, and crystallization tendency; solid dispersions and S(N)EDDS approaches are effective for reducing recrystallization and photodegradation risks [38,39,40]. Sensory correction in this group is best achieved through acidic citrus profiles and “top-note” modulation with moderate sweetening [41]. These measures align with the morning window objective: creating a stable aglycone exposure plateau without dose escalation, consistent with the concentration–time PBPK curves.

In the evening LiverGuard Tea/shot (aqueous format), anchor glycosides—Rutin and Diosmin—fall into the high-TPSA/low-lipophilicity region, indicating poor passive permeation and limited solubility, thus requiring enhanced solubilization and degradation protection. For Rutin, low solubility/permeability and risk of deglycosylation under harsh conditions are principal concerns; these are mitigated by inclusion complexes (e.g., sulfobutyl ether-β-cyclodextrin) and enzymatic polyglucosylation, both improving solubility and stability [42,43]. For Diosmin, low solubility and photolability are critical; complexes with cyclodextrins and nanosystems (including nanosponges), combined with UV-barrier packaging, are justified [44,45]. Technological best practices involve short hot-water contact times and minimization of oxidative losses, aligning with the evening window objective: moderate peaks with prolonged target coverage.

In the postprandial HDL-Chews (chewable format), the anchor component Genistein combines moderate lipophilicity with relatively low TPSA; this property profile supports rapid release from chewable/film matrices and achievement of the target coverage metric in the 0–6 h interval. Key risks for this isoflavone are thermal degradation and oxidation, both mitigated by protein/polysaccharide carriers, antioxidant buffers, and reduced water activity in solid dispersions [46]. Sensory optimization here is achieved through berry-acid profiles and physical taste-masking techniques, validated in food and pharmaceutical applications [41]. From an engineering standpoint, the dose corridor established in (minimally sufficient genistein dose ≈ 160 mg for %T>IC50 ≥ 50% at 1 μM, and the option of exposure enhancement by rutin co-administration without genistein escalation) directly translates into requirements for chewable matrix mass, release rate, and flavor-masking schemes.

Integration of these considerations into a unified design framework is captured in Figure 3 and Table 1. Figure 3 visualizes the distribution of anchor actives in logP–TPSA coordinates (with point size proportional to MW), illustrating why evening glycosides require aqueous forms with solubilization and UV protection, morning flavonols require glassy capsules with photo- and antioxidant protection, and the postprandial isoflavone requires rapidly releasing chewable carriers. Table 1 consolidates MW/logP/TPSA descriptors, key stability risks, recommended carriers/matrices, and predicted sensory profiles with supporting references; as a unifying technological thesis, a comprehensive review of polyphenol encapsulation and spray-drying applicability in food systems is cited [47]. Together, this “probabilistic profile → PBPK exposure → physicochemical descriptor → technological design” chain provides a reproducible roadmap from in silico predictions to engineering specifications, pending experimental validation.

**Table 1:** Predicted stability and sensory profiles (in silico, based on ranked_hits.csv/portfolio.csv). Values correspond to programmatically generated outputs (see Jupyter notebook); references are matched per compound.

Form	Active Compound	MW	logP	TPSA	Key Stability Risks	Recommended Carrier/Matrix	Predicted Flavor Profile	Literature Refs.
Morning	Baicalin	446.36	1.0	187.0	pH-dependent glycosidic hydrolysis; photodegradation; oxidation of phenolic groups	Microencapsulation (maltodextrin/arabic gum); light-impermeable packaging	bitter, herbal, astringent	[30,31]
Morning	Myricetin	318.24	1.6	151.6	Poor solubility; oxidation/darkening in aqueous systems; pH and temperature sensitivity	Liposomal/self-emulsifying systems; cyclodextrins; co-solvents (PEG)	bitter-astringent, floral-herbal	[32,33,34]
Morning	Luteolin	286.24	2.4	111.1	Low solubility; photolability; crystallization tendency	Solid dispersions; co-crystals; self-emulsifying phospholipid preparations (LSEPP)	bitter, astringent	[38,39]
Evening	Rutin	610.52	1.6	269.4	Poor aqueous solubility; acid-catalyzed hydrolysis (deglycosylation) under harsh conditions; low permeability	Micronization + surfactants; rutin polyglucosylation; hard gelatin capsule	mild bitterness, astringent	[42,43]
Evening	Diosmin	608.54	2.2	269.4	Poor solubility and permeability; photolability	Nanosuspensions; cyclodextrin complexes; tablets with antioxidants and UV-barrier packaging	bitter, slightly citrus/astringent	[44,45]
Postpr	Genistein	270.24	2.0	90.9	Thermal degradation; oxidation; isoflavone tautomeric/isomeric transitions	Protein-based matrices (soy isolate), film/chewable forms; antioxidant buffers	bitter, bean-/nut-like	[41,46]

## 3. Discussion

The fully in silico workflow presented here for multitarget nutraceutical screening in MASLD demonstrates the advantages of an integrated architecture over the single-target strategies and linear biomarker-driven selections that still predominate in the literature [1]. Unlike traditional approaches that focus on the “best” molecule according to a single metric, our framework deliberately constructs product concepts in which nuclear receptors (FXR, PPAR-α/γ, THR-β), lipogenesis/cholesterologenesis enzymes (ACC1, FASN, DGAT2, HMGCR), and transport/regulatory nodes (LIPG, FABP4) are addressed in a balanced manner—fully consistent with current understanding of multi-target interventions in metabolic liver disease [13,48].

A key methodological result is the coupling of abstraction layers, from target-specific activity probabilities to system-level indices, and finally to technological feasibility. At the predictive level, the ensemble (Random Forest, XGBoost, CatBoost) with stochastic stacking, Murcko-/time-split partitioning, and leakage control produced robustly calibrated probabilities *p*(active): mean ROC-AUC = 0.834 (median = 0.840; range = 0.750–0.916) in cross-validation and 0.840 (median = 0.832; range = 0.758–0.956) on independent tests. The strongest results were achieved for FABP4 (0.956), THR-β (0.930), and HMGCR (0.882), while DGAT2 (0.758), FXR (0.765), and PPAR-γ (0.774) were more challenging. Model concordance was high (integral Kendall’s W = 0.829; mean pairwise Jaccard = 0.716), and calibration was confirmed by Brier scores (mean = 0.094; median = 0.088; range = 0.018–0.150) and near-perfect Hosmer–Lemeshow *p*-values (≥0.995). These properties are fundamental: they elevate probabilistic predictions from “surrogate” values to engineering-grade inputs—sufficient for accurate dose calculation and exposure-window design.

At the chemico-biological level, structural mapping at a Tanimoto threshold of 0.20 yielded one dominant “core” cluster (32 compounds) and five singletons (six clusters in total), whereas clustering on *p*(target) vectors produced 12 MOA-clusters (sizes 9, 6, 5, 5, 3, 2, 2, and five singletons). This dual-layer approach ensured that despite partial scaffold convergence, the functional and pharmacological diversity of the prioritized pool remained sufficient for downstream formulation design. The probabilistic heat map revealed consistent patterns of co-activation (notably, LIPG with THR-β and ACC1) and no strictly “narrow” selectivity profiles (max *p* ≥ 0.80, second-best ≤ 0.40) within the current set. This highlights the nontrivial “structure → mechanism” relationship: distinct modes of multi-target activity can emerge from the same structural motif. The probabilistic heat map revealed consistent patterns of co-activation (notably, LIPG with THR-β and ACC1) and no strictly “narrow” selectivity profiles (max *p* ≥ 0.80, second-best ≤ 0.40) within the current set. Detectable “breaks” in local SAR (Δ*p* ≥ 0.40 at Tanimoto ≥ 0.80) occurred sporadically at baseline thresholds and became more evident under relaxed criteria, opening opportunities for fine-grained intra-cluster optimization without altering overall topology.

Transition to product-level solutions demonstrated practical feasibility across three concepts, where TA and TA_weighted served as multi-target coverage criteria and PBPK modeling set the engineering objective of %T>IC50 within the relevant chrono-windows. The morning HepatoBlend formula (baicalin + myricetin; alternatively baicalin + luteolin) produced a hepatic exposure plateau relevant to FXR/PPAR-α cascades (C_max_ ≈ 2.4 μM at t_max_ ~2 h, maintained ≥1 μM for 6–7 h). Crucially, the target metric was calculated not by arithmetic summation of concentrations but via per-target effects E(t) = C(t)/(IC_50_ + C(t)) (Hill n = 1), aggregated across components and targets. The evening aqueous formulation LiverGuard Tea/shot (rutin + diosmin) provided a smoothed profile (C_max_ ≈ 1.35–1.40 μM at t_max_ ~2 h) with maintenance above 1 μM up to ~6 h, consistent with an aqueous matrix and slower glycoside absorption. The postprandial chewable HDL-Chews (genistein) achieved the target 0–6 h coverage at a minimally sufficient dose of ~160 mg (C_max_ ~1.2 μM; 100 mg peaked ~0.75 μM; 150–200 mg reached ~1.15–1.50 μM). Exposure augmentation with rutin (200–300 mg alongside 100 mg genistein) elevated total coverage (C_max_ ≈ 1.2–1.45 μM) without escalating the genistein dose. Placement of these decisions on the logP–TPSA map illustrates that carrier and stabilization choices follow directly from the physicochemical coordinates of “anchor” actives: morning flavonols justify glassy polysaccharide matrices with photo- and antioxidant protection; evening glycosides require aqueous forms with solubilization (e.g., cyclodextrin complexes, polyglucosylation) and UV-barrier packaging; the postprandial isoflavone requires rapidly releasing chewable/film carriers. Final compositions were curated from the auto-portfolio with restrictions on structural/MOA diversification, compliance with regulatory limits, and sensory acceptability.

At present, chrono-specificity is implemented through distinct exposure objectives for morning, evening, and postprandial windows. In future iterations, Ka(t) and Cl(t) will be modeled as circadian functions to reflect diurnal enzyme activity and intestinal permeability rhythms.

The workflow inherently supports personalization: pathway weights in TA_weighted can be adjusted to reflect patient phenotypes (e.g., emphasis on anti-dyslipidemic vs. anti-inflammatory axes), while PBPK parameters can be tuned to body mass, liver condition, diet, and chronotype—making the approach compatible with digital nutrigenomics platforms and the broader concept of personalized nutrition [49]. Accordingly, the simulated %T>IC50 and C_max_ metrics should be regarded as computational indicators of exposure sufficiency, to be experimentally validated in subsequent in vitro and pilot PK studies (see Table S1). At the same time, limitations are significant: the study was entirely in silico, primary in vitro data sources were heterogeneous, and assumptions of IC_50_ comparability, simplified PBPK models (enzyme clearance, transporters, presystemic metabolism, microbiota effects, conjugation/deglycosylation), and matrix effects may bias bioavailability estimates. Future iterations of the PBPK model will therefore expand beyond the one-compartment Bateman framework to incorporate interindividual variability, transporter-mediated fluxes, microbiota-driven metabolism, and conjugation pathways, thereby improving the physiological realism of exposure predictions. Interindividual variability (enzyme polymorphisms, microbiome composition, circadian rhythms) further widens exposure ranges at fixed doses. Regulatory differences in acceptable daily intakes must be considered when adjusting doses and selecting technological enhancers consistent with food status.

A stepwise validation trajectory appears practically relevant: (i) ex vivo/in vitro confirmation of priority interactions and parallel solubility/stability studies in intended matrices; (ii) pharmacokinetic mini-studies in volunteers to recalibrate PBPK parameters considering formulation, carrier, and food effects; (iii) pilot intervention trials with digital monitoring of metabolic markers (lipid profile dynamics, liver fat proxies)—a “model-to-evidence” pathway enabling confirmation or adaptation of engineering specifications. Finally, the pipeline is transferable: it can be scaled to other natural libraries and related metabolic conditions by reconfiguring the target panel, pathway weights, and PBPK criteria. In the context of MASLD heterogeneity, such a multi-target logic offers more realistic prospects for clinical efficacy than single-target strategies [1,13,48], and aligns with the agenda of personalized nutrition [49].

In addition to predictive performance and formulation feasibility, toxicological and ADMET aspects were systematically evaluated within the pipeline (Appendix A). All shortlisted compounds passed GRAS/Novel Food and DSSTox safety filters, showed no PAINS structural alerts, and exhibited acceptable in silico profiles for absorption, distribution, metabolism, and toxicity. Known flavonoid metabolism routes—phase II glucuronidation and sulfation—are expected to dominate systemic clearance, producing conjugates with residual antioxidant or signaling activity. Potential herb–drug interactions, particularly CYP450 inhibition and P-glycoprotein modulation, will be addressed in forthcoming in vitro microsomal and transporter assays. Collectively, these observations indicate that the proposed multitarget actives fall within a favorable safety domain for nutraceutical development.

### 3.1. Limitations and Future Work

The present study is entirely in silico and employs a screening-level, one-compartment Bateman model to translate calibrated probabilities into exposure-linked dose suggestions. Accordingly, the %T>IC50 and C_max_ values should be regarded as first-order approximations rather than definitive pharmacokinetic measurements.

Future work will focus on a staged validation program designed to bridge these computational results with experimental data:

(i) In vitro biochemical and cell-based assays to confirm target engagement and direction of effect for priority compounds;
(ii) Ex vivo human liver microsome studies to characterize Phase I/II metabolism, conjugation, and clearance;
(iii) Small-scale in vivo pharmacokinetic studies using food-grade formulations to benchmark simulated exposures against observed C_max_/AUC values.

Future in vitro and ex vivo validation will specifically examine whether simulated exposure metrics (%T>IC50, C_max_) correspond to observed pharmacodynamic thresholds. These validation stages will refine clearance and bioavailability parameters, strengthen model calibration, and support the optimization of chrono-specific product concepts. A detailed validation roadmap is provided in Table S1 (Supplementary Materials).

A full physiologically based PBPK model incorporating multi-compartment kinetics will be developed during the experimental validation stage (Table S1). For baicalin specifically, future PK mini-studies will quantify plasma and hepatic exposure to replace literature-based analog inference (baicalein) with compound-specific parameters (Table S1).

Future PBPK refinements will also integrate circadian variation in hepatic metabolism and intestinal absorption to improve the physiological realism of chrono-specific formulations.

### 3.2. Preliminary Benchmarking Against Literature PK Data

To assess plausibility, the simulated hepatic exposure peaks (C_max_) were compared with plasma pharmacokinetic data from human studies of representative flavonoids. For genistein, oral doses of 30–300 mg yielded plasma C_max_ ≈ 0.9–6.7 µM (Ullmann et al., 2005, [50]), showing a clear dose–response trend. For rutin, reported quercetin plasma C_max_ ≈ 0.5–1.3 µM (t_max_ 6–8 h) after 200 mg, reflecting delayed glycoside absorption (Erlund et al., 2000, [51]). For baicalin, human PK studies mainly report its aglycone baicalein with plasma C_max_ ≈ 1–2 µM (t_max_ 2 h) following 200–300 mg oral doses. The corresponding simulated hepatic C_max_ values—~1.35 µM (rutin), ~2.4 µM (baicalin/baicalein), and ~1.2 µM (genistein)—fall within the same order of magnitude as reported plasma exposures, confirming that the screening-level model yields physiologically plausible estimates.

## 4. Materials and Methods

### 4.1. Assembly of the Protein Target Panel and Construction of the Biological Database

This study was grounded in a deliberately curated panel of ten protein targets spanning key nodes of the pathogenesis of metabolic-associated steatotic liver disease (MASLD) (Table 2). The sensing layer is represented by the nuclear receptors Farnesoid X receptor (FXR), Peroxisome proliferator-activated receptor alpha (PPAR-α), Peroxisome proliferator-activated receptor gamma (PPAR-γ), and Thyroid hormone receptor beta (THR-β), which integrate hormonal and nutrient cues and mediate transcriptional regulation of lipid metabolism [11,12]. The catalytic arm comprises the rate-limiting enzymes of de novo lipogenesis and cholesterologenesis—Acetyl-CoA carboxylase 1 (ACC1), Fatty acid synthase (FASN), Diacylglycerol O-acyltransferase 2 (DGAT2), and HMG-CoA reductase (HMGCR)—that act as “throttles” on triglyceride and cholesterol biosynthetic fluxes [52]. The transport-regulatory layer is represented by Endothelial lipase (LIPG) and Fatty acid-binding protein 4 (FABP4), which connect lipid transport with inflammatory and stress-signaling cascades [53,54]. The clinical relevance of this panel is supported by independent proteomic studies (raw and processed mass-spectrometry data available in PRIDE/ProteomeXchange: PXD011345 and PXD008762), which demonstrate marked differential expression of most of these proteins in MASLD and their contribution to multifactorial models explaining a substantial proportion of MRI-PDFF variance [55,56,57]. It is important to underscore that all subsequent results were obtained exclusively in silico; no de novo wet-lab experiments were performed in this work.

Quantitative interaction data for small molecules against each target were obtained from ChEMBL (version 33), a resource compliant with the FAIR principles and providing standardized aggregation of bioanalytical records [58]. For each target, identified by its target_chembl_id, we retrieved in vitro results with IC_50_ values (nanomolar scale), accompanied by structural information and experimental metadata; queries were executed via the REST API, and only records with numeric activity values were included in the working extract. All data were consolidated into a unified dataframe; records lacking structural annotations or numeric results were discarded.

Subsequent database preparation involved multi-step structure standardization. For every compound we generated canonical SMILES and an InChIKey, removed counter-ions, performed tautomer normalization, and adjusted protonation states to pH 7.4. Compounds matching on the first InChIKey block together with an identical tautomer signature were treated as duplicates; their IC_50_ values were aggregated by the median to mitigate the influence of outliers. To unify the target label and cast the task in binary form, we applied a 1 μM threshold: IC_50_ ≤ 1 μM was encoded as “active,” and larger values as “inactive,” while preserving original numeric values for subsequent calibration of probabilistic predictions [29]. Replicate measurements for a given compound–target pair were used to estimate the relative standard error (RSE); if RSE exceeded 15%, the label was treated as censored and excluded from the training core, thereby reducing the risk of propagating unreliable data into the model. For downstream system-level aggregation and coverage calculations in the TA/TA_weighted metrics, we employed uniform per-target probability thresholds of *p*(active) = 0.50, ensuring comparable “credit” of targets across compounds and their combinations.

The final step in database assembly aligned structures with regulatory and toxicological registries relevant to functional food design. For each InChIKey, we cross-walked to a CAS Registry Number and checked for entries in FDA GRAS and EFSA Novel Food lists; in the absence of matches, we conducted additional screening in DSSTox and Tox21 to detect potential toxicophore signals. Based on these checks, each candidate was assigned a status of food-grade, experimental gap, or red-flag; only candidates without red flags were considered in subsequent in silico triage and formulation.

**Table 2:** Panel of MASLD-relevant targets and weighted contributions to total activity (TA_weighted).

Target	Biological Role	Reference Metric	Key Literature References
THR-β	Regulation of basal metabolic rate and lipid metabolism	0.882	[59]
FXR	Bile acid and lipid metabolism regulator	0.736	[11]
FABP4	Intracellular fatty acid transport protein	0.875	[54]
PPAR-γ	Adipogenesis, glucose and lipid metabolism	0.922	[31]
PPAR-α	Fatty acid oxidation, lipoprotein metabolism	0.924	[12]
HMGCR	Cholesterol biosynthesis rate-limiting enzyme	0.918	[60]
ACC1	Fatty acid synthesis rate-limiting enzyme	0.917	[61]
DGAT2	Triacylglycerol synthesis	0.558	[62]
LIPG	HDL metabolism (endothelial lipase)	0.678	[63]
FASN	Synthesis of long-chain fatty acids	0.879	[64]

### 4.2. Descriptor Generation and Predictive Modeling

Following the assembly of a high-quality database of chemical structures with binary activity labels, we generated molecular descriptors and trained predictive models to estimate the probability that each compound interacts with the selected protein targets. Structural representations were computed with RDKit [65]. Each molecule was treated as a graph (vertices—atoms with formal charges; edges—covalent bonds with encoded orders) and then subjected to canonical aromatization and enumeration of implicit hydrogens. This normalization ensured proper handling of π-delocalization, conjugation, and valence—prerequisites for reliable topological and physicochemical indices. Substructural diversity was encoded using extended Morgan fingerprints with radius 2 and a bit vector length of 2048, enabling capture of second-order atomic environments and pairwise comparison via the Tanimoto coefficient. This configuration (Morgan r = 2, nBits = 2048, sim = Tanimoto) was fixed in the project settings and used through clustering and chemical-space mapping [66]. In parallel, we computed a set of continuous physicochemical descriptors (molecular weight, counts of H-bond donors/acceptors, Crippen logP, TPSA, rotatable bonds, elemental composition, and others), providing a complementary representation to detect subtle substitution effects that purely bit-based encodings may miss [66].

The resulting feature matrix underwent multi-stage curation: removal of near-zero-variance features (constant/quasi-constant), suppression of multicollinearity among continuous descriptors (for |r| > 0.95 we retained the feature with higher mutual information with the activity label), and robust scaling of the continuous block (median centering and interquartile-range scaling). This preprocessing reduced the influence of heavy-tailed distributions and outliers typical of chemical data, stabilizing training under class imbalance and source heterogeneity.

The first-level ensemble comprised three learners spanning distinct bias–variance trade-offs: Random Forest (RF), XGBoost, and CatBoost [67,68,69]. RF (500 trees) provided a resilient baseline and noise attenuation on sparse features; XGBoost enhanced performance by modeling nonlinear interactions between continuous descriptors and fragment bits; CatBoost employed ordered boosting to mitigate target leakage and overfitting on rare motifs. All models accounted for class imbalance via inverse-frequency weighting of the positive (active) class. Base-model predictions were combined at the second level using logistic meta-regression in a stacked-generalization framework [70]. The meta-model was trained strictly on the out-of-fold (OOF) probability matrix to prevent validation leakage and yielded interpretable weights for the RF/XGBoost/CatBoost contributions. For well-calibrated probabilistic outputs, we applied isotonic calibration to align predicted *p*(active) with empirical frequencies; calibration status of the base learners is recorded in the final quality summary table (“bases_calibrated”) and is reflected in subsequent reliability analyses of the probabilities [70].

**Figure 4:**
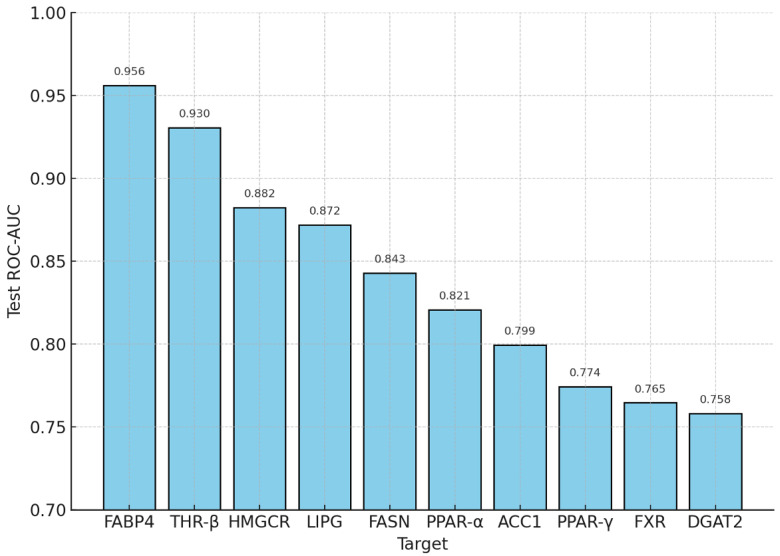
Predictive performance of target-specific models.

Hyperparameter optimization for the base models and the meta-regressor employed Bayesian optimization via the Tree–Parzen Estimator (TPE) [7], with ROC-AUC as the objective metric under scaffold-aware cross-validation stratified by Bemis–Murcko scaffolds. This protocol constrained leakage of closely related chemotypes across folds and yielded a more conservative estimate of generalization; the use of scaffold stratification is consistent with inclusion of Murcko scaffolds in the project pipeline configuration [66]. The result was a calibrated stacked ensemble delivering stable, target-specific probabilities of activity across all ten targets, thereby providing a quantitative foundation for downstream multitarget triage and hierarchical ranking of candidates [71] (Figure 4).

### 4.3. Post-Processing and Integral Metrics

Once calibrated probabilities of activity had been obtained for each compound across the ten targets (Table 3), the next step involved their aggregation into integral indices. These indices were designed to simultaneously capture the strength of multi-target coverage while maintaining a controllable level of structural diversity.

As a “soft” metric, we employed the weighted sum.

$$s=\sum_{t}=w_{t}p_{t}$$

TA_weighted, where *p_t_* denotes the predicted probability of activity for target t, and the weights *w_t_* are defined in the pipeline configuration (weights_path). In the absence of explicit weights, the aggregation reduces to a mean-weighted sum across targets. This scheme is embedded within the post-processing module and serves as one of the principal scoring functions for candidate ranking.

For stage-wise filtering, we applied the integer-valued metric total activity (TA), equal to the number of targets for which pt exceeds the per-target threshold. Thresholds were fixed uniformly at 0.50 for all targets, consistent with the calibrated probability scale and ensuring an interpretable “active/inactive” cut-off at the target level. Portfolio selection was then governed by the following rule: TA ≥ 3 (high priority), TA = 1–2 (medium priority), TA = 0 (low priority). Candidates were ordered hierarchically by TA, followed by the main weighted score (weighted_sum/main_score), and TA_weighted served as a tie-breaker. This hierarchy prevents situations in which moderate probabilities across many targets would artificially outperform a narrower but stronger coverage, while retaining sensitivity to calibrated effect magnitudes.

**Figure 5:**
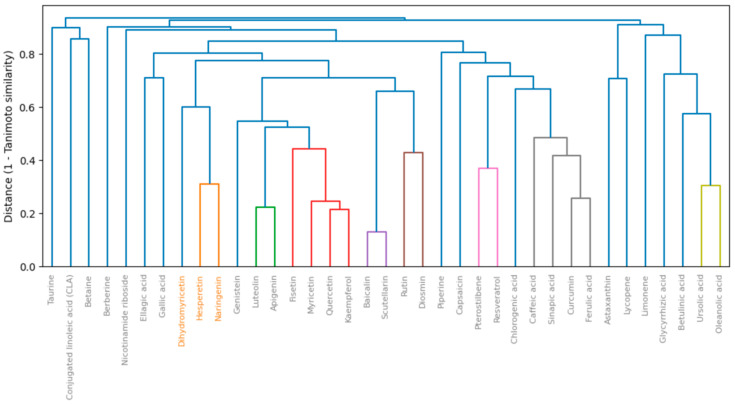
Chemical space dendrogram (Tanimoto/Morgan r = 2, nBits = 2048). Colors indicate distinct structural clusters.

To preserve chemical space breadth, structural clustering was performed using the Tanimoto coefficient computed on Morgan fingerprints (radius = 2, n_bits = 2048) [66] (Figure 5). The clustering cut-off was determined automatically via silhouette maximization. The parameters employed in the report were: fp = morgan, radius = 2, n_bits = 2048, sim = tanimoto, auto_cut = true, selected threshold = 0.20. Diagnostic dendrograms and internal diversity indices confirmed a robust clustering solution, with the priority pool reaching an integral diversity index (ID) of 0.72—indicative of enhanced diversity relative to the input library. In degenerate cases, the algorithm employed stable fallback strategies (threshold shifts toward maximum similarity, kNN-based communities, MST-cut), preventing the emergence of either a single giant cluster or a fully fragmented space.

To link topological proximity with biological profiles, cluster-level averages of activity probabilities were computed and Shannon entropy was used as a measure of selectivity: low entropy values reflected pharmacophore specialization, whereas high values indicated multi-target potential. Special attention was given to “activity breaks”—abrupt drops in pt among structurally similar molecules. These were detected using thresholds Tanimoto ≥ 0.8 and Δ*p* ≥ 0.4, as defined in the configuration (break_gap). Blocks of co-activation (e.g., FXR–PPAR-α–LIPG) and local discontinuities helped to highlight substructures driving the binding profile.

Reliability of prioritization was assessed through inter-model concordance. The Wender–Kendall coefficient (W) demonstrated high rank agreement across Random Forest, XGBoost, and CatBoost—both averaged across the pool and at the per-target level (W = 0.83 overall; range 0.56–0.92 across targets) [72]. Complementary Jaccard analysis of top-N lists confirmed consensus-based rather than “monopolistic” selection. Time-split resampling validated the robustness of rankings under shifting temporal boundaries of training data [73]. These validation procedures, coupled with established decision-tree ensemble best practices, provided confidence in the stability of prioritization.

### 4.4. In Silico Formulation Design

At the final stage of the in silico workflow, the results of predictive modeling and multi-target ranking were translated into the practical context of functional food product development through the calculation of compositions and formulation characteristics. The baseline parameter for design was the minimum simulated daily dose (MSD), derived from a PBPK model that integrated intestinal absorption, portal vein transport, and hepatic tissue distribution (C_(hep)_) for each lead compound and its corresponding chrono-application window. Default parameter values were Ka = 0.8 h^−1^ and Cl = 0.1 L·h^−1^·kg^−1^ for reference compounds, scaled according to molecular weight and logP. Volume of distribution (Vd) was estimated using the empirical relationship Vd = 0.6 + 0.02 × logP (L·kg^−1^). Representative parameter sets for all simulated compounds are summarized in Table S2, Input parameters for screening-level PBPK simulations. A comparison between simulated hepatic C_max_ and reported human plasma C_max_ values for representative flavonoids is provided in Table S3, demonstrating order-of-magnitude agreement between the screening-level model and independent pharmacokinetic data [74]. The engineering criterion was defined as target-time coverage across the priority proteins of a given window; the primary design threshold was set at 70%. In cases where pharmacokinetically constrained exposure limited attainment, a conservative tolerance of 50–60% was accepted, provided that technological strategies for enhancing bioavailability were explicitly justified [28]. This approach prevented artificial inflation of dosages and enabled rational recourse to formulation strategies that improve C_(hep)_ without exceeding acceptable dietary intake limits.

It should be noted that this PBPK implementation represents a simplified one-compartment Bateman-type model intended for screening-level exposure estimation, not a full physiologically based PBPK simulation. The model approximates intestinal absorption and hepatic distribution using empirical scaling from molecular weight and logP, providing indicative rather than definitive pharmacokinetic estimates.

The computed MSD values were systematically cross-checked against EFSA/FDA regulatory limits for food-grade substances to exclude scenarios in which modeled hepatic exposure would necessitate surpassing permitted daily intakes. For candidates that exceeded such thresholds, design decisions assigned them to an adjunctive (adjuvant) role within composite formulations rather than as single-molecule bases. Recipe optimization was posed as a linear programming problem: the objective function maximized the aggregate activity index (combination of TA and TA_weighted) subject to constraints on total portion mass, adherence to chrono-administration windows (morning/evening/postprandial), structural diversity (minimum Tanimoto distance among included leads), and regulatory compliance. This ensured theoretical “stitching” of daily multi-target coverage across the MASLD pathogenic network through complementary time windows.

Carrier selection and protection strategies for active compounds were guided by physicochemical properties and literature evidence on polyphenol processability. For moderately lipophilic aglycones, glass-forming polysaccharide matrices (e.g., maltodextrin DE ≈ 10, inulin) were evaluated for their ability to slow autoxidation and provide controlled release. For more polar glycosides, protein–polysaccharide complexes with hydrophobic pockets were considered, theoretically reducing oxidative degradation and improving storage stability [75]. In cases of poor solubility and absorption-limited exposure, phytosomal encapsulation (phospholipid complexes) was modeled, drawing on published evidence for substantial increases in oral bioavailability (e.g., quercetin up to ~5.2×) [28]. Such values were used as benchmarks for scenario-based recalculation of C_(hep)_, without altering the original target prioritization order.

Application of this framework to the three prototype product concepts revealed typical constraints and their corresponding solutions. In the morning window (HepatoBlend morning), simulated profiles reached ~54–59% coverage for myricetin- and luteolin-based variants at moderate doses—an “engineering-acceptable” level but below the 70% target, thus requiring exposure-enhancing interventions such as increased proportion of glassy matrices and/or hybrid carriers incorporating partial lipid phases. In the evening window (LiverGuard Tea evening), baseline coverage was ~37%, indicating the need either for dose escalation within permissible ranges or the addition of a synergist with a complementary profile (e.g., enhanced FXR/PPAR-α or LIPG coverage) within the allowable portion mass. In the postprandial window (HDL-Chews postpr), a mismatch was observed between high predicted *p*(active) for the target and zero coverage at the baseline dose, which was interpreted as an absorption/first-pass limitation. Here, phytosomal encapsulation, co-administration with lipid phases, and cross-scenario testing of intake relative to meals were justified strategies [28,32]. A consolidated composition and the calculated indices for all three product concepts are summarized in Table 4.

**Figure A1:**
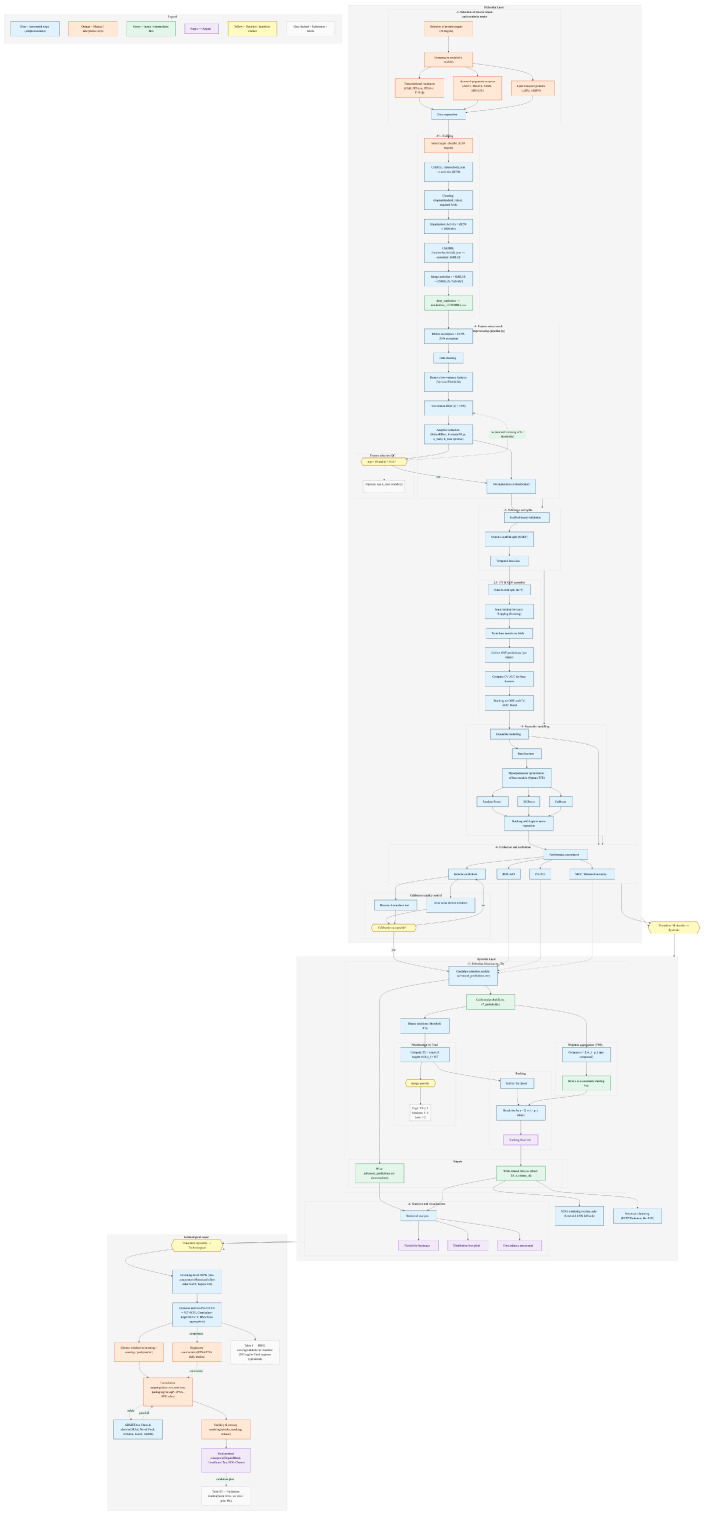
Overview of the fully in silico pipeline integrating molecular, systemic, and technological design layers.

In summary, in silico formulation design enabled the translation of probabilistic “target × compound” profiles into practical product specifications: defining MSD and administration regimen within chrono-logic, aligning with regulatory admissibility, selecting carriers and exposure-enhancement technologies to achieve target coverage, and maintaining structural diversity within the portfolio. Future stages of research will involve experimental implementation of these concepts and validation of the predicted properties under laboratory and clinical conditions. A detailed schematic of the complete workflow is provided in Appendix A (Figure A1).

## 5. Conclusions

This work presents an end-to-end, fully in silico workflow for multitarget nutraceutical screening aimed at mitigating metabolic-associated steatotic liver disease (MASLD). The central idea was to align, within a single computational trajectory, three traditionally disjoint design layers: (i) the molecular layer—predicting activity against a validated ten-target MASLD panel; (ii) the systems layer—aggregating target-specific probabilities into integral indices that account for pathway contributions; and (iii) the technological layer—translating biological and PBPK considerations into early-stage formulation specifications (carriers, packaging, sensory). This “through-line” architecture minimizes the gap between high-level efficacy criteria and practical constraints on manufacturability that are typical of nutraceutical development.

At the molecular level, we assembled a high-quality biological database, curated target-specific activity records, and generated an expanded set of structural descriptors (bit-based and continuous). A three-learner ensemble (Random Forest, XGBoost, CatBoost) with second-level stacking and isotonic calibration produced calibrated probabilities *p*(active) per target. Murcko- and time-split partitions, class-imbalance corrections, and leakage control during stacking yielded stable quality estimates: mean ROC-AUC in cross-validation 0.834 (median 0.840; range 0.750–0.916), and on independent tests 0.840 (median 0.832; 0.758–0.956). The strongest test results were observed for FABP4 (0.956), THR-β (0.930), and HMGCR (0.882), and the most challenging for DGAT2 (0.758), FXR (0.765), and PPAR-γ (0.774). Inter-model ranking agreement remained high (integral Kendall–Wender W = 0.829; mean pairwise Jaccard 0.716), while probability calibration was supported by aggregate Brier scores (mean 0.094; median 0.088; range 0.018–0.150) and near-unity HL-*p* values (≥0.995). Practically, calibration is what converts “raw” model scores into frequency-like quantities, thereby legitimizing downstream engineering operations (thresholding, weighting, PBPK calculations).

System-level aggregation was implemented through two complementary metrics. The stepwise metric TA (the count of targets with *p*(active) above a calibrated threshold) supplied a discrete criterion for stage-wise filtering and served as the primary ranker. The “soft” metric TA_weighted (weighted sum of probabilities, with weights reflecting relative pathway contributions to pathogenesis) differentiated compounds with similar TA values by incorporating the priorities of regulatory sensors, lipogenic enzyme nodes, and transport axes. Together, these metrics imparted interpretability (how many and which targets are covered) and sensitivity to network importance—attributes that are especially pertinent to a multitarget MASLD strategy.

The structural map of chemical space confirmed that the prioritized pool retained sufficient breadth: Tanimoto clustering of Morgan fingerprints (r = 2; 2048 bits) with silhouette-based auto-cut yielded a dominant core (n = 32) plus a periphery of five singletons (six clusters in total). The internal diversity index exceeded that of the source library, and “activity breaks” (sharp drops in *p*(active) at high topological proximity) helped outline structural determinants of selectivity and multitarget behavior. This layer served not only as an interpretive aid but also as a design constraint to prevent over-convergence of formulations onto narrow pharmacophoric motifs downstream.

PBPK modeling constituted the key “bridge” between biology and technology. Hepatic exposure forecasts (concentration–time curves) and %T>IC50 calculations within the relevant chrono-windows (“morning”, “evening”, “postprandial”) set engineering criteria for minimum daily dose and timing of administration. These numerical anchors harmonized intended multitarget action with realistic usage regimens. Notably, for the postprandial window the model identified a minimally sufficient genistein dose of ~160 mg to achieve %T>IC50 ≥ 50% over 0–6 h and revealed the benefit of exposure “augmentation” via rutin co-administration—findings that immediately translate into portion-mass requirements, release matrices, and sensory masking. For the morning concept, total Chep(t) reached C_max_ ≈ 2.4 μM at t_max_ ~2 h with maintenance ≥1 μM for ~6–7 h, whereas the evening aqueous form produced a smoothed profile (C_max_ ≈ 1.35–1.40 μM, t_max_ ~2 h) with levels ≥ 1 μM up to ~6 h.

At the technological level, we formalized in silico rules that connect physicochemical descriptors (MW, logP, TPSA) with stability risks (photo- and oxidative degradation, acid–base hydrolysis, recrystallization), carrier selection (glassy polysaccharides, emulsifying/phospholipid systems, cyclodextrins, nanosuspensions), packaging requirements, and sensory models (masking bitterness/astringency, dosing acidic and aromatic notes). The logP–TPSA visualization (Figure 3) for the “anchor” actives, together with the consolidated risk/carrier table (Table S4), rendered this layer reproducible and auditable: the same fallback properties used in the portfolio were applied, and recommendations were grounded in consensus literature across compound classes. While not a substitute for experiments, such early “technological filtration” rationally narrows option space before laboratory work begins.

The outcome is three product concepts, all derived in silico and logically aligned with MASLD pathobiology and chronopharmacology: HepatoBlend (morning powder focused on FXR/PPAR-α with an early-day exposure plateau), LiverGuard Tea/shot (evening aqueous form based on polar glycosides with moderate peaks and a long tail), and HDL-Chews (postprandial chewable targeting LIPG/FABP4, with an option for exposure enhancement via rutin). In each case, dose, matrix, packaging, and sensory specifications were derived coherently from calibrated per-target probabilities, aggregated metrics, PBPK profiles, and physicochemical predictors.

The proposed methodology offers several fundamental advantages. First, it ensures end-to-end traceability of decisions—from ranked_hits.csv/portfolio.csv and configuration files to final design parameters. Second, it is personalizable: TA_weighted weights, %T>IC50 targets, chrono-windows, and sensory constraints can be adapted to phenotype, lifestyle, and preferences. Third, it is scalable: the same stages and criteria apply to other classes of natural compounds and related metabolic conditions, given appropriate reconfiguration of target panels and weights.

We nonetheless emphasize the limitations of the approach. All outputs—from activity predictions to PBPK profiles and technological recommendations—are computational; no laboratory validation was performed here. A stepwise validation agenda is therefore required: (i) in vitro/ex vivo confirmation of priority interactions and parallel solubility/stability studies in the intended matrices; (ii) pharmacokinetic mini-studies to recalibrate PBPK parameters considering matrix and fed/fasted state; (iii) stability and sensory testing for the nominated carriers and packaging; and (iv) pilot clinical studies with digital monitoring of metabolic markers. Regulatory daily intake limits and evidence requirements vary across jurisdictions and must be accounted for when adjusting doses and selecting food-grade enhancers.

This study presents a fully in silico and reproducible proof-of-concept pipeline. Experimental validation (in vitro target engagement, ex vivo microsomal assays, and small-scale pharmacokinetic studies) constitutes the next stage to confirm the simulated exposure metrics and the predicted multitarget profiles.

In conclusion, we show that an integrated in silico pipeline—from calibrated probabilities across a MASLD target panel to PBPK-oriented doses and technological specifications—can serve as a pragmatic engine for generating rational, multitarget nutraceutical concepts. Its value lies not in predicting an “ideal” molecule but in assembling a viable formulation whose key risks and requirements are pre-resolved. This increases the likelihood of downstream experimental success and accelerates the “hypothesis → prototype → validation” cycle. Future work will focus on externalizing to independent libraries, cross-validating PBPK parameters in humans, refining the sensory model with target consumer panels, and adapting target weights to MASLD clinical subtypes—steps expected to enable personalized products with reproducible profiles of efficacy and acceptability.

## Figures and Tables

**Table 3:** Model performance metrics.

Cv_Auc_ Std	Test_Roc_Auc	Test_Pr_ Auc	Test_ Accuracy	Test_ Balanced_ Accuracy	Test_ Precision	Test_Recall	Test_F1	Test_Mcc	Calibration	T_Opt
0.0	0.956	0.938	0.875	0.873	0.857	0.857	0.857	0.746	{‘note’: ‘bases_calibrated’}	0.497
0.0	0.93	0.922	0.767	0.783	1.0	0.565	0.722	0.614	{‘note’: ‘bases_calibrated’}	0.5
0.0	0.882	0.944	0.851	0.781	0.899	0.909	0.904	0.571	{‘note’: ‘bases_calibrated’}	0.507
0.0	0.872	0.974	0.902	0.746	0.948	0.942	0.945	0.482	{‘note’: ‘bases_calibrated’}	0.917
0.0	0.843	0.934	0.833	0.727	0.878	0.915	0.896	0.483	{‘note’: ‘bases_calibrated’}	0.752
0.0	0.821	0.745	0.758	0.757	0.757	0.726	0.741	0.515	{‘note’: ‘bases_calibrated’}	0.467
0.0	0.799	0.848	0.694	0.745	0.919	0.596	0.723	0.464	{‘note’: ‘bases_calibrated’}	0.625
0.0	0.774	0.739	0.715	0.709	0.786	0.557	0.652	0.44	{‘note’: ‘bases_calibrated’}	0.708
0.0	0.765	0.535	0.75	0.54	0.75	0.091	0.162	0.2	{‘note’: ‘bases_calibrated’}	0.361
0.0	0.758	0.45	0.95	0.778	0.583	0.583	0.583	0.557	{‘note’: ‘bases_calibrated’}	0.257

Values are presented for the baseline 100 mg scenario (prior to optimization); optimized regimens are detailed in Section 2.3.

**Table 4:** PBPK-derived pharmacokinetic coverage for prioritized compounds. Baseline (100 mg) scenario represents the pre-optimization simulation; optimized regimen values correspond to final product-specific formulations described in Section 2.3. All %T>IC50 metrics are calculated per-target using the Hill n = 1 model and aggregated across components and chrono-windows.

Product	Window	Target	*p*_Mean	pct_T_gt_IC_50_ (Baseline 100 mg Scenario, Prior to Optimization)
HDL_Chews_postpr	postpr	CHEMBL5080	0.9166666666666666	0.0
HepatoBlend_morning_luteolin	morning	CHEMBL2083	0.291507311396635	58.75000000000001
HepatoBlend_morning_luteolin	morning	CHEMBL239	0.0833333333333333	58.75000000000001
HepatoBlend_morning_luteolin	morning	CHEMBL3351	0.5833333333333333	58.75000000000001
HepatoBlend_morning_myricetin	morning	CHEMBL2083	0.291507311396635	53.74999999999999
HepatoBlend_morning_myricetin	morning	CHEMBL239	0.14583333333333331	53.74999999999999
HepatoBlend_morning_myricetin	morning	CHEMBL3351	0.5833333333333333	53.74999999999999
LiverGuardTea_evening	evening	CHEMBL1947	0.6272597242443584	36.66666666666667
LiverGuardTea_evening	evening	CHEMBL2047	0.1686898326408873	36.66666666666667
LiverGuardTea_evening	evening	CHEMBL235	0.45	36.66666666666667

## Data Availability

All curated datasets, prediction outputs, configuration files, and post-processing scripts used in this study are provided as Supporting Information (CSV, JSON, and Python files) and are also available via GitHub (https://github.com/4kumax/masld-flavonoid-screen, accessed on 14 October 2025). These materials are sufficient to reproduce the reported results. Raw activity data can be retrieved directly from the ChEMBL API (https://www.ebi.ac.uk/chembl, accessed on 14 October 2025). Additional details are available from the corresponding author upon reasonable request.

## Supplementary Materials

The following supporting information can be downloaded at: https://www.mdpi.com/article/10.3390/molecules30214159/s1, Table S1: Planned staged validation roadmap for priority flavonoids and targets; Table S2: Input parameters for screening-level PBPK simulations; Table S3: Benchmarking of simulated vs. reported pharmacokinetic parameters for representative flavonoids; Table S4: Consolidated risk/carrier matrix for prioritized compound classes.

## Abbreviations

The following abbreviations are used in this manuscript:

MASLD	Metabolic dysfunction-associated steatotic liver disease
TA	Total activity
PBPK	Physiologically based pharmacokinetic
NAFLD	“Non-alcoholic fatty liver disease”
T2D	Type 2 diabetes
FFA	Free fatty acid
MOA	Mechanism-of-action

## Appendix A

In this study, we developed and validated a fully in silico pipeline for the multi-target screening of nutraceutical candidates for MASLD, which integrates:

(i) the formation of a biological target panel underpinned by systems biology data (FXR, PPAR-α/γ, THR-β receptors; ACC1, FASN, DGAT2, HMGCR enzymes; transport-regulatory proteins LIPG, FABP4);
(ii) extraction and standardization of quantitative bioactivity data from ChEMBL followed by chemical curation;
(iii) generation of molecular descriptors (RDKit functional groups and extended Morgan fingerprints);
(iv) ensemble predictive modeling (Random Forest, XGBoost, CatBoost) with meta-level logistic regression (stacked generalization) and probability calibration;
(v) post-processing and multi-target ranking incorporating target weights;
(vi) translation of the results into technological design via PBPK modeling of liver exposure and preliminary assessment of formulation stability/sensory properties.

A schematic of the overall workflow is presented in Figure A1.

Automated processes (blue) correspond to Python modules and scripted routines; manual or interpretive steps (orange) denote expert validation and decision-making. Layer transitions are marked by labeled dividers. The diagram also references ADMET/tox filters, regulatory nodes, Table 4 (Baseline vs. Final Regimen), and Table S1 (Validation Roadmap). All workflows were implemented in Python 3.10 using RDKit 2023.03.2, scikit-learn 1.4.2, XGBoost 2.0.3, and CatBoost 1.2.3.

## References

[1] Eslam M., Sanyal A.J., George J. (2020). MAFLD: A Consensus-Driven Proposed Nomenclature for Metabolic Associated Fatty Liver Disease. Gastroenterology.

[2] Chan W.-K., Chuah K.-H., Rajaram R.B., Lim L.-L., Ratnasingam J., Vethakkan S.R. (2023). Metabolic Dysfunction-Associated Steatotic Liver Disease (MASLD): A State-of-the-Art Review. J. Obes. Metab. Syndr..

[3] Chrysavgis L., Cholongitas E. (2024). From NAFLD to MASLD: What does it mean?. Expert Rev. Gastroenterol. Hepatol..

[4] Song R., Li Z., Zhang Y., Tan J., Chen Z. (2024). Comparison of NAFLD, MAFLD and MASLD characteristics and mortality outcomes in United States adults. Liver Int..

[5] Targher J., Byrne C.D., Tilg H. (2024). MASLD: A systemic metabolic disorder with cardiovascular and malignant complications. Recent Adv. Clin. Pract..

[6] Li Y., Yang P., Ye J., Xu Q., Wu J., Wang Y. (2024). Updated mechanisms of MASLD pathogenesis. Lipids Health Dis..

[7] Wang D., Miao J., Zhang L., Zhang L. (2024). Research advances in the diagnosis and treatment of MASLD/MASH. Ann. Med..

[8] Obaseki E., Adebayo D., Bandyopadhyay S., Hariri H. (2024). Lipid droplets and fatty acid-induced lipotoxicity: In a nutshell. FEBS Lett..

[9] Hammerich L., Tacke F. (2023). Hepatic inflammatory responses in liver fibrosis. Nat. Rev. Gastroenterol. Hepatol..

[10] Friedman S.L., Neuschwander-Tetri B.A., Rinella M., Sanyal A.J. (2018). Mechanisms of NAFLD development and therapeutic strategies. Nat. Med..

[11] Lefebvre P., Cariou B., Lien F., Kuipers F., Staels B. (2009). Role of Bile Acids and Bile Acid Receptors in Metabolic Regulation. Physiol. Rev..

[12] Kersten S. (2014). Integrated Physiology and Systems Biology of PPARα. Mol. Metab..

[13] Pawlak M., Lefebvre P., Staels B. (2015). Molecular Mechanism of PPARα Action and Its Impact on Lipid Metabolism, Inflammation and Fibrosis in NAFLD. J. Hepatol..

[14] Long J., Xu Y., Zhang X., Wu B., Wang C. (2024). Role of FXR in the development of NAFLD and intervention strategies of small molecules. Arch. Biochem. Biophys..

[15] Todisco S., Santarsiero A., Convertini P., De Stefano G., Gilio M., Iacobazzi V., Infantino V. (2022). PPAR Alpha as a Metabolic Modulator of the Liver: Role in the Pathogenesis of Nonalcoholic Steatohepatitis (NASH). Biology.

[16] Zhu Z., Zhang X., Pan Q., Zhang L., Chai J. (2023). In-depth analysis of de novo lipogenesis in non-alcoholic fatty liver disease: Mechanism and pharmacological interventions. Liver Res..

[17] Calle R.A., Amin N.B., Carvajal-Gonzalez S., Ross T.T., Bergman A., Aggarwal A., Crowley C., Rinaldi A., Mancuso J., Aggarwal N. (2021). ACC inhibitor alone or co-administered with a DGAT2 inhibitor in patients with non-alcoholic fatty liver disease: Two parallel, placebo-controlled, randomized phase 2a trials. Nat. Med..

[18] O’Farrell M., Duke G., Crowley R., Buckley D., Martins E.B., Bhattacharya D., Friedman S.L., Kemble G. (2022). FASN inhibition targets multiple drivers of NASH by reducing steatosis, inflammation and fibrosis in preclinical models. Sci. Rep..

[19] Li T., Yan H., Geng Y., Shi H., Li H., Wang S., Wang Y., Xu J., Zhao G., Lu X. (2019). Target genes associated with lipid and glucose metabolism in non-alcoholic fatty liver disease. Lipids Health Dis..

[20] Attal N., Sullivan M.T., Girardi C.A., Thompson K.J., McKillop I.H. (2021). Fatty acid binding protein-4 promotes alcohol-dependent hepatosteatosis and hepatocellular carcinoma progression. Transl. Oncol..

[21] Wang X., Li M., Yu F., Hou L., Cao R., Zhang L., Xie J., Wang F., Huang J. (2024). Astragalus polysaccharide inhibits lipogenesis in HFD-fed mice by suppressing LIPG via upregulation of UDPG. Adv. Compos. Hybrid. Mater..

[22] Tan P., Jin L., Qin X., He B. (2022). Natural flavonoids: Potential therapeutic strategies for non-alcoholic fatty liver disease. Front. Pharmacol..

[23] Pisonero-Vaquero S., Gonzalez-Gallego J., Sanchez-Campos S., Garcia-Mediavilla M.V. (2015). Flavonoids and Related Compounds in Non-Alcoholic Fatty Liver Disease Therapy. Curr. Med. Chem..

[24] Van De Wier B., Koek G.H., Bast A., Haenen G.R.M.M. (2017). The potential of flavonoids in the treatment of non-alcoholic fatty liver disease. Crit. Rev. Food Sci. Nutr..

[25] Nguyen T., Le H., Quinn T.P., Nguyen T., Le T.D., Venkatesh S. (2021). GraphDTA: Predicting drug–target binding affinity with graph neural networks. Bioinformatics.

[26] Chou W.-C., Lin Z. (2023). Machine learning and artificial intelligence in physiologically based pharmacokinetic modeling. Toxicol. Sci..

[27] Saldívar-González F.I., Aldas-Bulos V.D., Medina-Franco J.L., Plisson F. (2022). Natural product drug discovery in the artificial intelligence era. Chem. Sci..

[28] Boonpawa R., Moradi N., Spenkelink B., Rietjens I.M.C.M., Punt A. (2015). Use of physiologically based kinetic (PBK) modeling to study interindividual human variation and species differences in plasma concentrations of quercetin and its metabolites. Biochem. Pharmacol..

[29] Williamson G., Manach C. (2005). Bioavailability and bioefficacy of polyphenols in humans. II. Review of 93 intervention studies. Am. J. Clin. Nutr..

[30] Panche A.N., Diwan A.D., Chandra S.R. (2016). Flavonoids: An overview. J. Nutr. Sci..

[31] Tontonoz P., Spiegelman B.M. (2008). Fat and beyond: The diverse biology of PPARγ. Annu. Rev. Biochem..

[32] Riva A., Ronchi M., Petrangolini G., Bosisio S., Allegrini P. (2019). Improved Oral Absorption of Quercetin from Quercetin Phytosome^®^, a New Delivery System Based on Food Grade Lecithin. Eur. J. Drug Metab. Pharmacokinet..

[33] Xing J., Chen X., Zhong D. (2005). Stability of baicalin in biological fluids in vitro. J. Pharm. Biomed. Anal..

[34] Feng Z., Zhou J., Shang X., Kuang G., Han J., Lu L., Zhang L. (2017). Comparative research on stability of baicalin and baicalein administrated in monomer and total flavonoid fraction form of Radix scutellariae in biological fluids in vitro. Pharm. Biol..

[35] Gaber D.M., Nafee N., Abdallah O.Y. (2017). Myricetin solid lipid nanoparticles: Stability assurance from system preparation to site of action. Eur. J. Pharm. Sci..

[36] Khatamian N., Motavalizadehkakhky A., Homayouni Tabrizi M., Mehrzad J., Zhiani R. (2023). Preparation and characterization of the myricetin-loaded solid lipid nanoparticles decorated with folic acid-bound chitosan and evaluation of its antitumor and anti-angiogenic activities in vitro and in vivo in mice bearing tumor models. Cancer Nano.

[37] Sims K.R., He B., Koo H., Benoit D.S. (2020). Electrostatic Interactions Enable Nanoparticle Delivery of the Flavonoid Myricetin. ACS Omega.

[38] Alshehri S., Imam S.S., Altamimi M.A., Hussain A., Shakeel F., Elzayat E., Mohsin K., Ibrahim M., Alanazi F. (2020). Enhanced Dissolution of Luteolin by Solid Dispersion Prepared by Different Methods: Physicochemical Characterization and Antioxidant Activity. ACS Omega.

[39] Ansari M.J., Alshetaili A., Aldayel I.A., Alablan F.M., Alsulays B., Alshahrani S., Alalaiwe A., Ansari M.N., Rehman N.U., Shakeel F. (2020). Formulation, characterization, in vitro and in vivo evaluations of self-nanoemulsifying drug delivery system of luteolin. J. Taibah Univ. Sci..

[40] Salawi A. (2022). Self-emulsifying drug delivery systems: A novel approach to deliver drugs. Drug Deliv..

[41] Coupland J.N., Hayes J.E. (2014). Physical approaches to masking bitter taste: Lessons from food and pharmaceuticals. Pharm. Res..

[42] De Gaetano F., Pastorello M., Pistarà V., Rescifina A., Margani F., Barbera V., Ventura C.A., Marino A. (2024). Rutin/Sulfobutylether-β-Cyclodextrin as a Promising Therapeutic Formulation for Ocular Infection. Pharmaceutics.

[43] González-Alfonso J.L., Poveda A., Arribas M., Hirose Y., Fernández-Lobato M., Ballesteros A.O., Jiménez-Barbero J., Plou F.J. (2021). Polyglucosylation of rutin catalyzed by cyclodextrin glucanotransferase from *Geobacillus* sp.: Optimization and chemical characterization of products. Ind. Eng. Chem. Res..

[44] Ai N., Ma Y., Wang J., Li Y. (2014). Preparation, Physicochemical Characterization and In-Vitro Dissolution Studies of Diosmin-Cyclodextrin Inclusion Complexes. Iran. J. Pharm. Res..

[45] Anwer M.K., Ahmed M.M., Aldawsari M.F., Iqbal M., Kumar V. (2022). Preparation and Evaluation of Diosmin-Loaded Diphenylcarbonate-Cross-Linked Cyclodextrin Nanosponges for Breast Cancer Therapy. Pharmaceuticals.

[46] Rostagno M.A., Palma M., Barroso C.G. (2004). Pressurized liquid extraction of isoflavones from soybeans. Anal. Chim. Acta.

[47] Gharsallaoui A., Roudaut G., Chambin O., Voilley A., Saurel R. (2007). Applications of spray-drying in microencapsulation of food ingredients: An overview. Food Res. Int..

[48] Ma K., Saha P.K., Chan L., Moore D.D. (2006). Farnesoid X receptor is essential for normal glucose homeostasis. J. Clin. Investig..

[49] Ordovás J.M., Ferguson L.R., Tai E.S., Mathers J.C. (2018). Personalised nutrition and health. Br. Med. J..

[50] Ullmann U., Metzner J., Frank T., Cohn W., Riegger C. (2005). Safety, tolerability, and pharmacokinetics of single ascending doses of synthetic genistein (Bonistein) in healthy volunteers. Adv. Ther..

[51] Erlund I., Kosonen T., Alfthan G., Mäenpää J., Perttunen K., Kenraali J., Parantainen J., Aro A. (2000). Pharmacokinetics of quercetin from quercetin aglycone and rutin in healthy volunteers. Eur. J. Clin. Pharmacol..

[52] Rong S., Xia M., Vale G., Wang S., Kim C.-W., Li S., McDonald J.G., Radhakrishnan A., Horton J.D. (2024). DGAT2 inhibition blocks SREBP-1 cleavage and improves hepatic steatosis by increasing phosphatidylethanolamine in the ER. Cell Metab..

[53] Broedl U.C., Jin W., Rader D.J. (2004). Endothelial lipase: A modulator of lipoprotein metabolism upregulated by inflammation. Trends Cardiovasc. Med..

[54] Furuhashi M., Hotamisligil G.S. (2008). Fatty acid-binding proteins: Role in metabolic diseases and potential as drug targets. Nat. Rev. Drug Discov..

[55] PXD011345 (ProteomeCentral). https://proteomecentral.proteomexchange.org/cgi/GetDataset?ID=PXD011345.

[56] PXD008762 (PRIDE Project). https://www.ebi.ac.uk/pride/archive/projects/PXD008762.

[57] Govaere O., Hasoon M., Alexander L., Cockell S., Tiniakos D., Ekstedt M., Schattenberg J.M., Boursier J., Bugianesi E., Ratziu V. (2023). A proteo-transcriptomic map of non-alcoholic fatty liver disease. Nat. Metab..

[58] Mendez D., Gaulton A., Bento A.P., Chambers J., de Veij M., Félix E., Magariños M.P., Mosquera J.F., Mutowo P., Nowotka M. (2019). ChEMBL: Towards direct deposition of bioassay data. Nucleic Acids Res..

[59] Brent G.A. (2012). Mechanisms of thyroid hormone action. J. Clin. Investig..

[60] Istvan E.S., Deisenhofer J. (2001). Structural mechanism for statin inhibition of HMG-CoA reductase. Science.

[61] Wakil S.J., Abu-Elheiga L.A. (2009). Fatty acid metabolism: Target for metabolic syndrome. J. Lipid Res..

[62] Yen C.L., Stone S.J., Koliwad S., Harris C., Farese R.V. (2008). Thematic review series: Glycerolipids. DGAT enzymes and triacylglycerol biosynthesis. J. Lipid Res..

[63] Yu J.E., Han S.Y., Wolfson B., Zhou Q. (2018). The role of endothelial lipase in lipid metabolism, inflammation, and cancer. Histol. Histopathol..

[64] Smith S. (1994). The animal fatty acid synthase: One gene, one polypeptide, seven enzymes. FASEB J..

[65] Landrum G. RDKit: Open-Source Cheminformatics Software. https://www.rdkit.org.

[66] Rogers D., Hahn M. (2010). Extended-Connectivity Fingerprints. J. Chem. Inf. Model..

[67] Breiman L. (2001). Random Forests. Mach. Learn..

[68] Chen T., Guestrin C. XGBoost: A scalable tree boosting system. Proceedings of the 22nd ACM SIGKDD International Conference on Knowledge Discovery and Data Mining.

[69] Prokhorenkova L., Gusev G., Vorobev A., Dorogush A.V., Gulin A. (2018). CatBoost: Unbiased boosting with categorical features. NeurIPS.

[70] Wolpert D.H. (1992). Stacked generalization. Neural Netw..

[71] Bergstra J., Yamins D., Cox D. Making a science of model search: Hyperparameter optimization in hundreds of dimensions for vision architectures. Proceedings of the 30th International Conference on Machine Learning.

[72] Probst P., Wright M.N., Boulesteix A.L. (2019). Hyperparameters and tuning strategies for random forest. WIREs Data Min. Knowl. Discov..

[73] Sheridan R.P. (2013). Time-split cross-validation as a method for estimating the goodness of prospective prediction. J. Chem. Inf. Model..

[74] Li L., Gao H., Lou K., Luo H., Hao S., Yuan J., Liu Z., Dong R. (2021). Safety, tolerability, and pharmacokinetics of oral baicalein tablets in healthy Chinese subjects: A single-center, randomized, double-blind, placebo-controlled multiple-ascending-dose study. Clin. Transl. Sci..

[75] Díaz-Montes E. (2023). Wall Materials for Encapsulating Bioactive Compounds via Spray-Drying: A Review. Polymers.

