# Supplementary material for: Using Machine Learning for the Discovery and Development of Multitarget Flavonoid-Based Functional Products in MASLD
Source: Molecules. 2025 Oct 22;30(21):4159. doi: 10.3390/molecules30214159 (PMC12609199; doi:10.3390/molecules30214159)
Supplement: Supplementary file 1 [file molecules-30-04159-s001.zip › Table S4.pdf]

Table S4. Consolidated risk/carrier matrix for prioritized compound classes. Key physicochemical risks and corresponding formulation strategies ensuring stability, solubility, and sensory compatibility of multitarget flavonoid-based nutraceuticals.

Compound Class	Key Stability Risks	Recommended Carriers / Formulation Systems	Notes / References
**Flavones (e.g., luteolin, apigenin)**	Poor aqueous solubility; photo- and oxidative degradation; recrystallization	Cyclodextrin inclusion complexes; phospholipid or liposomal systems; glassy polysaccharide matrices	Stable under mildly acidic pH; protect from light exposure; use light-proof packaging
**Isoflavones (e.g., genistein, daidzein)**	pH-sensitive stability (alkaline > acidic); low bioavailability; precipitation in aqueous phase	Nanosuspensions; zein nanoparticles; lecithin-based liposomes; polymeric micelles	Enhanced absorption with lipid carriers; avoid strong alkalis
**Flavonols (e.g., quercetin, myricetin, rutin)**	Oxidative degradation; metal-ion complexation; bitterness/astringency	β- or HP-β-Cyclodextrin inclusion complexes; phospholipid emulsions; microencapsulation with antioxidants (ascorbic acid)	Mask astringency with natural acids/aromas; protect from oxidation
**Phenolic acids (e.g., caffeic, ferulic)**	pH-sensitive stability; oxidative coupling/polymerization under alkaline or oxidative conditions	Maltodextrin or gum arabic encapsulation; protein–polysaccharide films	Suitable for aqueous or dry systems; avoid prolonged heating
**Glycosides (e.g., baicalin, hesperidin)**	Hydrolysis in acidic or β-glucuronidase-rich environments; poor solubility	Cyclodextrin inclusion complexes; emulsifying biopolymers; nanosuspensions	Stable under neutral pH; avoid strong acids; mild heating only
**Mixed formulations**	Component incompatibility; uneven dissolution or release	Sequential coating; layered granules; capsule segregation; differential release profiles (morning/evening)	Enables time-phased delivery and minimizes inter-component degradation
