# Supplementary material for: Using Machine Learning for the Discovery and Development of Multitarget Flavonoid-Based Functional Products in MASLD
Source: Molecules. 2025 Oct 22;30(21):4159. doi: 10.3390/molecules30214159 (PMC12609199; doi:10.3390/molecules30214159)
Supplement: Supplementary file 1 [file molecules-30-04159-s001.zip › Table S1.pdf]

Table S1. Planned staged validation roadmap for priority flavonoids and targets.
Abbreviations: ACC1 — acetyl-CoA carboxylase 1; DGAT2 — diacylglycerol O-acyltransferase 2; FASN — fatty acid synthase; HMGCR — HMG-CoA reductase; FXR/PPAR-α/PPAR-γ/THR-β — nuclear receptors; FABP4 — fatty acid-binding protein 4; LIPG — endothelial lipase.

**Stage**	**Objective**	**Representative assays / systems**	**Materials (priority actives)**	**Primary endpoints**	**Acceptance / readouts**	**Notes**
1. In vitro target engagement	Confirm predicted direction and potency vs enzyme/receptor panels	Enzymatic inhibition (ACC1, FASN, DGAT2, HMGCR); lipase assay (LIPG); reporter assays (FXR, PPAR-α/γ, THR-β)	Rutin, Baicalin, Myricetin, Luteolin, Genistein	IC_{50} / EC_{50}; efficacy vs control	Concordant direction of effect; potency ≤ 10× predicted IC_{50} threshold	Use standardized buffers; $n \geq 3$; include reference ligands
2. Ex vivo metabolism	Characterize Phase I/II metabolism & permeability	Human liver microsomes ± UDPGA/PAPS; Caco-2 transport assay	Same panel	Intrinsic clearance (Cl_int), metabolite ID; P_app	Parameter ranges sufficient to refine Ka, Cl, F	Links PBPK inputs to empirical data
3. Pilot PK (food-grade)	Benchmark exposure vs simulation	Open-label single-dose; standardized meal	Prototype formulations (spray-dried / phytosome)	C_{max}, T_{max}, AUC; tolerability	Observed / simulated C_{max} and AUC within 2–3×; safety acceptable	Morning / Evening / Post-prandial windows
4. Iterative refinement	Update model parameters	Refit Bateman model with measured Ka, Cl, F	—	Reduced bias of $\%T > IC_{50}$	Improved calibration (Brier ↓; HL $p > 0.05$)	Ensures data-driven parameterization
