# Supplementary material for: Using Machine Learning for the Discovery and Development of Multitarget Flavonoid-Based Functional Products in MASLD
Source: Molecules. 2025 Oct 22;30(21):4159. doi: 10.3390/molecules30214159 (PMC12609199; doi:10.3390/molecules30214159)
Supplement: Supplementary file 1 [file molecules-30-04159-s001.zip › Table S2.pdf]

Table S2. Input parameters for screening-level PBPK simulations.
Representative kinetic parameters used in the simplified one-compartment Bateman-type model for each prioritized compound.
Abbreviations: *Ka* — absorption rate constant; *Cl* — clearance; *Vd* — volume of distribution; *logP* — partition coefficient (octanol–water); *MW* — molecular weight; *TPSA* — topological polar surface area.

Compound	MW (Da)	logP	TPSA (Å²)	Ka (h^{-1})	Cl ($L \cdot h^{-1} \cdot kg^{-1}$)	Vd ($L \cdot kg^{-1}$)	Reference
Baicalin	446.36	1.0	187.0	0.8	0.10	0.62	[28, 29]
Myricetin	318.24	1.6	151.6	0.9	0.11	0.63	[28, 29]
Luteolin	286.24	2.4	111.1	1.0	0.12	0.65	[28, 29]
Rutin	610.52	1.6	269.4	0.6	0.09	0.63	[28, 29]
Diosmin	608.54	2.2	269.4	0.5	0.08	0.64	[28, 29]
Genistein	270.24	2.0	90.9	1.1	0.13	0.64	[28, 29, 32]
