# Supplementary material for: Using Machine Learning for the Discovery and Development of Multitarget Flavonoid-Based Functional Products in MASLD
Source: Molecules. 2025 Oct 22;30(21):4159. doi: 10.3390/molecules30214159 (PMC12609199; doi:10.3390/molecules30214159)
Supplement: Supplementary file 1 [file molecules-30-04159-s001.zip › Table S3.pdf]

Table S3. Benchmarking of simulated vs. reported pharmacokinetic parameters for representative flavonoids.
Abbreviations: C_{max} — maximum concentration; AUC — area under the curve; "Simulated" — output of the screening-level Bateman model (hepatic concentration, C_hep) used in this work; "Reported" — plasma concentrations from human oral PK studies. Note: Reported values are plasma levels and may include contributions of conjugated metabolites; simulated values represent hepatic exposure (C_hep). Direct numerical equality is not expected; we assess order-of-magnitude concordance.

**Compound (reported moiety)**	**Oral dose in literature (mg)**	**Reported C_{max} (μM, plasma)**	**Reported AUC (μM·h)**	**Simulated C_{max} (μM, hepatic)**	**Approx. ratio (Sim/Rep)**	**Key reference(s)**
Rutin → quercetin (plasma quercetin after oral rutin/quercetin)	~200	~0.5–1.3	~3.8–6.2	1.35	~1.0–2.7×	Erlund et al., 2000 (Eur J Clin Pharmacol) — similar C_{max} for aglycone vs rutinoside, longer t_{max} for rutin; numeric ranges summarized from the article and classical reviews. [51]
Baicalin (reported as baicalein in plasma)	200–300	~1.6–3.1*	—	2.4	~0.8–1.5×	Li et al., 2021 (Front Pharmacol): single-/multiple-dose baicalein PK in healthy subjects; day-1 C_{max} up to ~845 ng/mL (≈3.1 μM). [75]
Genistein (aglycone)	150–300	~3.0–6.7**	~5–15	1.2 (for 160 mg scenario in model)	~0.2–0.4× (vs 300 mg); ~0.7–1.3× (vs ≤150–200 mg)	Ullmann et al., 2005 (Mol Nutr Food Res/Arzneimittelforschung): single ascending doses 30–300 mg; C_{max} shows near dose-linearity ≤150 mg; mean C_{max} ≈1 800 ng/mL at 300 mg (≈6.7 μM). [50]

* Conversion for baicalein: 845 ng/mL ≈ 0.845 μg/mL; MW≈270 g/mol → ≈3.1 μM; lower end ~437 ng/mL ≈1.6 μM. Values from Li 2021 day-1 PK tables.
** Genistein numeric example: ~1 800 ng/mL at 300 mg ≈ 6.7 μM (MW≈270 g/mol). Near dose-linearity up to 150 mg implies ~1–3 μM bands for 150–200 mg.

Reported plasma concentrations reflect total circulating analyte (often dominated by conjugates), whereas simulated values correspond to hepatic exposure (C_hep) from a simplified screening model. Ranges compiled from the cited studies and authoritative reviews are used to assess order-of-magnitude plausibility rather than to claim identity between plasma and hepatic levels or between different doses and formulations.
